# Hydrogels in the Management of Peripheral Venous Disease: Bridging Material Design and Clinical Evidence

**DOI:** 10.3390/gels12090852

**Published:** 2026-09-19

**Authors:** Cristian-Adrian Silosi, Isabela Silosi, Mihai-Andrei Ruscu, Alexandra-Daniela Rotaru-Zavaleanu

**Affiliations:** 1 Discipline of Surgery, Faculty of Nursing, University of Medicine and Pharmacy of Craiova, 200349 Craiova, Romania; cristian.silosi@umfcv.ro; 2 Department of Surgery V, “Dr. Ștefan Odobleja” Emergency Military Clinical Hospital, 200749 Craiova, Romania; 3 SILOȘI Medical Center, 200347 Craiova, Romania; 4 Discipline of Immunology, Allergology and Clinical Immunology, University of Medicine and Pharmacy of Craiova, 200349 Craiova, Romania; 5 Department of Laboratory Medicine, Craiova County Emergency Clinical Hospital, 200642 Craiova, Romania; 6 Research Center for Microscopic Morphology and Immunology Studies, University of Medicine and Pharmacy of Craiova, 200349 Craiova, Romania; 7 Discipline of Epidemiology, University of Medicine and Pharmacy of Craiova, 200349 Craiova, Romania; alexandra.rotaru@umfcv.ro; 8 Research Center for Clinical and Experimental Medicine, University of Medicine and Pharmacy of Craiova, 200349 Craiova, Romania

**Keywords:** hydrogels, venous leg ulcer, chronic venous insufficiency, wound dressings, drug delivery, compression therapy, oxidative stress, matrix metalloproteinases, superficial venous thrombosis

## Abstract

Peripheral venous disease represents one of the most prevalent and most persistently undertreated chronic conditions in adult populations, with venous leg ulceration standing at the severe end of a spectrum that also includes symptomatic chronic venous insufficiency and superficial venous thrombosis. The venous wound microenvironment is shaped by mechanisms that are largely specific to this etiology, including ambulatory venous hypertension, erythrocyte extravasation with cutaneous iron deposition, sustained oxidative stress, proteolytic imbalance and fibroblast senescence. Hydrogels, as three-dimensional hydrophilic polymer networks with high water content, tunable mechanics and versatile payload capacity, offer an appealing platform for addressing several of these mechanisms simultaneously. The preclinical literature is correspondingly abundant. The clinical evidence base, however, remains thin and inconclusive, and the most rigorous synthesis available has been unable to establish whether hydrogel dressings outperform simpler alternatives in venous ulcer healing. This review examines that discrepancy rather than setting it aside. We summarize the physicochemical properties that determine hydrogel performance specifically under compression and in a heavily exudating wound, map hydrogel mechanisms of action onto venous pathobiology, survey therapeutic applications across the venous spectrum, including topical formulations for superficial venous thrombosis, and appraise the preclinical and clinical evidence with attention to why translation has stalled. We conclude by proposing design criteria for venous-specific hydrogel systems and methodological priorities for the trials needed to test them.

## 1. Introduction

### 1.1. The Clinical Burden of Peripheral Venous Disease

Peripheral venous disease of the lower limbs, used here to cover chronic venous disease and superficial venous thrombosis, spans a graded spectrum from telangiectasia to active ulceration, formalized in the CEAP classification [1,2]. Venous leg ulcers (VLUs) affect 1% to 2% of adults, rising to around 4% beyond 65 years, with age, female sex, obesity, family history, parity, immobility and prior deep vein thrombosis as established risk factors [1,3].

Care for chronic venous disease imposes a substantial burden on health systems, exceeding USD 3 billion annually in the United States and hundreds of millions of pounds within the United Kingdom National Health Service for leg ulceration alone [4,5,6]. Most of that cost is nursing time rather than devices or drugs, because these ulcers heal slowly, with 56 to 65% healed at 24 weeks even in compression-treated cohorts, recur commonly, and demand repeated dressing changes [7,8]. Exudate, pain and malodour erode quality of life in ways that healing rate captures poorly [2,9]. Superficial venous thrombosis (SVT), long treated as benign, is more prevalent than assumed and can extend into the deep system [10].

### 1.2. Limitations of Current Standard of Care

Compression therapy remains the cornerstone of management for advanced disease, supported by evidence that is unusually robust for the wound care field [1,2]. The landscape has been reshaped by the demonstration that early ablation of superficial reflux accelerates healing and reduces recurrence [11,12]. Venoactive pharmacotherapy adds symptomatic benefit in selected patients [13]. What has not advanced at the same pace is the primary wound contact layer. Dressings are applied beneath compression to manage exudate, protect the wound bed and facilitate autolytic debridement, yet the guiding evidence is sparse, and selection is driven largely by exudate volume, wound bed appearance and local formulary constraints rather than comparative outcome data [14,15].

Two bodies of literature have grown in parallel without meeting. Reviews of hydrogel wound dressings are numerous, but they pool venous, diabetic and pressure etiologies into a single chronic wound category and rarely engage with the constraint that in venous disease any dressing must function beneath sustained compression. Clinical reviews of venous ulcer management, conversely, treat dressings as an undifferentiated class and leave aside the material properties that distinguish one formulation from another. What is missing is a synthesis reading the material science against the venous-specific clinical evidence.

### 1.3. Rationale, Scope and Methodological Approach

The rationale for hydrogels here is both mechanistic and material. A crosslinked hydrophilic network retains water at fractions comparable to soft tissue while conforming to an irregular wound bed [16,17], and three variables govern its performance: swelling and vapor transmission, which determine tolerance of exudate; crosslinking chemistry, which governs residence time and release kinetics; and functionalization, which allows the network itself to act therapeutically [18,19,20,21]. Section 2 develops each in turn. Iron chelation belongs to the same logic and is directly suggested by the hemosiderin deposition characteristic of venous disease, although chelator-loaded hydrogels have so far been developed for other indications and for other purposes.

This review therefore has four aims: to summarize the features of venous wound pathobiology that constitute actionable targets for material design; to describe the polymer chemistries and functionalization strategies addressing those targets; to appraise the clinical evidence for hydrogel-based dressings in venous ulceration and related indications; and to identify the obstacles separating preclinical performance from proven clinical benefit. Coverage spans topical and locoregional applications across venous leg ulceration, symptomatic chronic venous insufficiency and superficial venous thrombosis, with deep vein thrombosis addressed only where local delivery is relevant.

A narrative format was chosen deliberately. The evidence spans in vitro characterization, animal models, small clinical series and a few randomized trials, and this heterogeneity of design, comparator and endpoint precludes quantitative synthesis while allowing material properties and clinical outcomes to be read against one another. Relevant literature was identified through MEDLINE via PubMed, Scopus and Web of Science, combining hydrogel terms with terms for venous disease, ulceration and wound dressings, emphasizing the past seven years and retaining earlier work where it remains the primary source. Searches were last updated in March 2026. We included English-language publications reporting hydrogel systems evaluated in venous indications, or reporting material properties relevant to performance under compression; conference abstracts were excluded. The manuscript follows the Scale for the Assessment of Narrative Review Articles (SANRA) [22]. The review is addressed primarily to the materials community: its aim is to specify what a hydrogel intended for venous disease must be designed and characterized to do, rather than to guide clinical dressing selection.

## 2. Hydrogel Fundamentals Relevant to the Venous Context

### 2.1. Network Architecture and Crosslinking

Hydrogels are three-dimensional networks of hydrophilic polymer chains capable of absorbing and retaining large volumes of water while resisting dissolution, a property conferred by crosslinks between chains [23]. The nature of those crosslinks is the single most consequential design decision in the venous setting, since it determines whether the material survives a week beneath a multilayer compression bandage on an ambulant patient, and it governs mechanical stability, degradation behavior and the release of any incorporated payload.

Chemically crosslinked networks rely on covalent bonds formed by radical polymerization, photo-initiated reactions, click chemistry or enzymatic coupling. They offer mechanical robustness and degradation behavior that can be programmed through the choice of hydrolysable or enzymatically cleavable linkages, although many covalent networks are effectively non-degradable over the timescale of a dressing change [24]. Their principal liability is the possible retention of unreacted monomer or initiator, which raises cytotoxicity concerns for application to a chronic wound with a compromised barrier [25].

Physically crosslinked networks rely on ionic interactions, hydrogen bonding, hydrophobic association, crystallite formation or host–guest recognition. They are generally produced under milder conditions and often permit encapsulation of labile biologics, but they are more susceptible to erosion under shear and to ion exchange with wound exudate. Calcium-crosslinked alginate is the instructive case, since exchange of Ca^2+^ for the Na^+^ present in exudate progressively destabilizes the network, and venous exudate is both abundant and ionically rich [16].

Dynamic covalent networks, built on imine, hydrazone, boronate ester or Diels–Alder linkages, occupy an intermediate position. Bonds form and reform under physiological conditions, which confers self-healing and injectability and, in the case of boronate esters, intrinsic responsiveness to the elevated pH of the chronic wound [19]. Dual-network and interpenetrating architectures pursue the same reconciliation by other means. In the canonical design, both networks are covalent, with sacrificial bonds in a rigid and brittle first network rupturing to dissipate energy while a soft, ductile second network preserves integrity [26]. Subsequent development has moved toward hybrid architectures combining covalent and physical crosslinks, and toward fully physically crosslinked variants in which reversible bonds provide dissipation together with self-recovery and fatigue resistance [27].

### 2.2. Classification by Polymer Origin

The polymers used to build these networks divide, conventionally, by origin, and the division has consequences that go beyond nomenclature. At the clinically translated end of the field sit the natural polymers: alginate, chitosan, hyaluronic acid, collagen and gelatin, cellulose derivatives and fibrin. What makes them attractive is that they carry biological information as well as providing structure. Alginate gels within seconds in the presence of divalent cations and takes up exudate efficiently, though the resulting network is mechanically weak and vulnerable to the ion exchange described above [16,28,29]. Chitosan brings antimicrobial and hemostatic activity of its own, and its cationic character helps it adhere to tissue surfaces, but it must be processed in acid or chemically modified before it will dissolve, and residual acidity is poorly tolerated on a wound that is already painful [30,31]. Hyaluronic acid intervenes directly in the signaling of tissue repair, although the direction of that intervention depends on chain length: long chains suppress inflammation, whereas the oligomers released as they break down are pro-inflammatory and pro-angiogenic [32,33]. Since depolymerization in the chronic wound proceeds through reactive oxygen species as well as endogenous hyaluronidase, crosslinking is less an enhancement than a prerequisite here. Collagen and gelatin, finally, supply the adhesion motifs that cells recognize, and the price is batch variability together with the sourcing, viral and prion safety documentation that animal-derived material entails.

Poly(vinyl alcohol) is worth singling out. Gelation by repeated freezing and thawing forms crystallite crosslinks and dispenses with chemical crosslinkers altogether, which simplifies the toxicological assessment considerably, although performance depends closely on degree of hydrolysis, molecular weight and freezing regime, so each formulation must be validated in its own right. Irradiation occupies an unusual dual position here, since gamma or electron beam exposure both crosslinks the network and sterilizes it in a single step, which is an attractive manufacturing simplification, while also altering the physical properties of an already freeze-thawed network in ways that require dose optimization [34,35,36].

Most current activity falls between these poles. Methacrylated gelatin and methacrylated hyaluronic acid, together with nanocomposites loaded with clays, metal or metal oxide nanoparticles, carbon-based fillers or drug-bearing micelles, attempt to keep biological recognition while acquiring mechanical and processing control. Complexity accumulates in proportion, since every added component enlarges both the characterization burden and the regulatory dossier. There is also a precedent worth recalling. Silver-containing dressings were developed on an antimicrobial rationale at least as persuasive as anything the nanocomposite literature now offers, yet randomized evaluation found no benefit for healing in venous ulceration [8]. Table 1 sets these classes against the performance requirements that matter in the venous setting.

### 2.3. Physicochemical Properties That Determine Venous Performance

A venous-specific review must diverge from the generic chronic wound literature at this point, because several properties conventionally presented as hydrogel strengths behave differently when the wound is a heavily exudative gaiter-area ulcer under sustained compression.

The first of these is fluid handling, and before it can be discussed at all a terminological ambiguity must be stated plainly. In wound care practice, the word hydrogel conventionally denotes an amorphous, moisture-donating gel formulated to rehydrate dry eschar and facilitate autolytic debridement. In materials science, it denotes any crosslinked hydrophilic network, a class that includes highly absorbent constructs with the opposite fluid behavior. The distinction matters because the prototypical venous ulcer is not dry. It is hyperexudative, and excess fluid pooling against the periwound skin produces maceration, wound enlargement, increased infection risk and pain [40,41]. That skin is poorly equipped to tolerate it: the gaiter area in advanced disease combines the woody induration of lipodermatosclerosis with epidermal atrophy, stasis dermatitis and atrophie blanche, so the tissue is simultaneously rigid and fragile [42]. What determines performance is therefore the equilibrium swelling ratio, the fluid handling capacity measured under compression rather than at rest, as specified for primary dressings in EN 13726, and the moisture vapor transmission rate of any backing layer. We argue that the mismatch between the moisture-donating design intent of classical amorphous hydrogels and the moisture-removal requirement of the typical venous ulcer is an underappreciated explanation for the modest clinical performance this class has shown in controlled evaluation, and that it directs attention toward systems engineered for net absorption, such as superabsorbent composites and hydrogel-impregnated foam constructs.

What the dressing does at its margins matters almost as much as what it does at its center. The periulcer skin in advanced chronic venous insufficiency is atrophic, hyperpigmented, indurated and frequently sensitized by years of topical exposure, so adhesion sufficient to maintain conformal contact under limb movement must coexist with removal that neither strips epidermis nor provokes pain. Catechol-functionalized systems inspired by mussel adhesive proteins, together with reversible and on-demand detachable adhesion chemistries, are the principal strategies under investigation [43,44,45].

**Figure 1:**
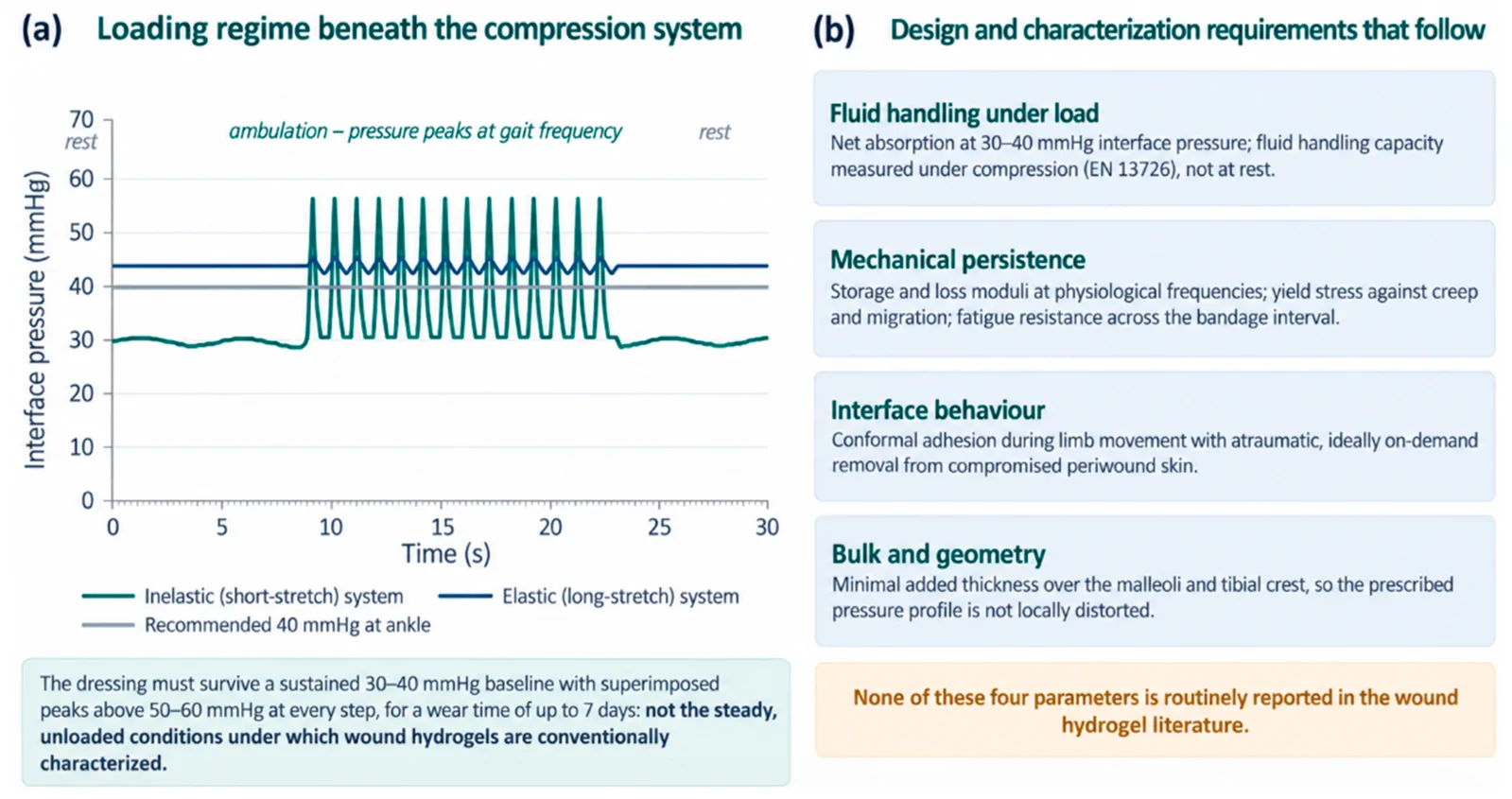
Loading regime imposed by compression therapy and the material characterization requirements that follow from it. (**a**) Schematic interface pressure profile beneath an inelastic, short-stretch system (teal) and an elastic, long-stretch system (blue) over a rest, ambulation and rest sequence, against the recommended 40 mmHg at the ankle (gray). The inelastic system combines a tolerable resting baseline of approximately 30 mmHg with transient peaks exceeding 50 to 60 mmHg at gait frequency, whereas the elastic system yields with calf contraction and delivers a higher, constant load without peaks. A dressing applied beneath compression must therefore withstand a sustained baseline with superimposed cyclic peaks for a wear time of up to 7 days, rather than the quasi-static unloaded conditions under which wound hydrogels are conventionally characterized. (**b**) The four design and characterization requirements derived from this regime: net fluid absorption measured under 30 to 40 mmHg interface pressure in accordance with EN 13726; storage and loss moduli at physiological frequencies, yield stress and fatigue resistance across the bandage change interval; conformal adhesion with atraumatic removal from compromised periwound skin; and minimal added bulk over bony prominences. None of these parameters is routinely reported in the wound hydrogel literature. Pressure values are illustrative, drawn from the ranges reported in [46,48].

Compression itself imposes a further set of demands that the materials literature has largely overlooked. Sub-bandage pressures of 30 to 40 mmHg at the ankle are recommended for healing, but the parameter that governs hemodynamic efficacy is stiffness rather than pressure alone [46,47]. Inelastic, short-stretch systems do not yield when the calf muscle contracts, so each step generates a transient interface pressure peak that may exceed 50 to 60 mmHg while resting pressure remains low enough to be tolerated overnight; elastic systems yield with contraction and produce no such peaks, at the cost of a higher and less well-tolerated resting load [46,48]. Since the inelastic systems are the ones that reduce ambulatory venous hypertension most effectively [48], the loading regime a dressing must actually survive is a sustained baseline with superimposed peaks at gait frequency rather than a steady 40 mmHg. The properties that follow are the storage and loss moduli across physiological frequencies, the yield stress governing whether the gel flows and migrates away from the wound bed, and fatigue resistance across a wear time that may extend to 7 days. These are rarely reported, since the wound hydrogel literature characterizes materials predominantly under quasi-static unloaded conditions, and their absence is a systematic weakness in the translational evidence. Figure 1 summarizes this loading regime and the characterization requirements that follow from it.

How long the network persists under those conditions has consequences that reach beyond the wound. Dressing change frequency in venous ulceration is dictated primarily by exudate saturation and by the compression regimen rather than by degradation of the primary contact layer. However, persistence sets the ceiling on what any regimen can achieve, because a hydrogel that loses mechanical integrity, migrates, or disintegrates before the bandage is due for renewal forces an earlier change regardless of clinical need. Since nursing time dominates the cost structure of venous ulcer care, a formulation that reliably matches the wear time of the compression system it sits beneath may deliver health economic benefit even without demonstrable acceleration of healing. The argument is seldom made explicitly and deserves formal evaluation, since it implies that the appropriate endpoint for some hydrogel systems is cost per healed ulcer rather than time to healing.

Three further requirements are practical rather than mechanical, and each is routinely underestimated. Cytocompatibility is conventionally assessed under ISO 10993-5 against healthy murine fibroblast lines such as L929, whereas the cells that will actually encounter the material include senescent fibroblasts and dysregulated macrophages with altered susceptibility [49,50]. Sterilization interacts with network chemistry throughout, and the interaction is neither uniform nor predictable from first principles [51]. Irradiation may induce additional crosslinking or chain scission depending on the polymer and dose: gamma treatment of methacrylated gelatin increases stiffness, reduces pore size and slows biodegradation while impairing the sol–gel transition, whereas autoclaving and ethylene oxide reduce stiffness without altering degradation [52], and aqueous alginate degrades exponentially at doses as low as 10 kGy [51]. Ethylene oxide is doubly problematic, since residues are absorbed and retained by hydrated matrices and the treatment can itself raise crosslink density and depress swelling [51]. Collagen and gelatin dressings are consequently produced aseptically as a matter of routine [53], which frequently leaves aseptic processing as the only viable route for bioactive formulations. Stability is the last of these requirements and the least studied. Water loss through the primary packaging, syneresis and exposure to freeze–thaw cycling during distribution all bear on whether a formulation survives to the point of use in community and home care, where the majority of venous ulcer management occurs, yet shelf-life behavior under realistic distribution conditions is essentially absent from the wound hydrogel literature.

### 2.4. Functionalization Strategies

Beyond the passive functions described above, the network can be made to act therapeutically, and three routes dominate in practice, although the underlying release mechanisms are more finely divided than this grouping suggests [54]. Physical entrapment of a payload within the mesh is the simplest, giving release governed by diffusion and by degradation of the network. It suits small molecules but offers limited control, since a drug that is small relative to the mesh size escapes rapidly, whereas release slows dramatically once the two dimensions approach one another [54]. Affinity-based retention is the second route, whether through covalent tethering of bioactive moieties to the polymer backbone, antioxidant phenolics, iron-chelating groups or peptide sequences among them, or through the electrostatic and hydrophobic associations that retain charged and amphiphilic payloads without a covalent bond [54,55]. Both localize activity and prevent systemic exposure, at the cost of a more demanding synthesis and characterization. Stimulus-responsive linkages constitute the third route, releasing payload in response to a feature of the wound itself, most commonly elevated pH, protease activity or reactive oxygen species concentration, and thereby coupling delivery to the pathological state rather than to elapsed time [56]. This last route is of particular interest for labile biological payloads, since the network protects them from degradation until the trigger is met [56]. Each of these routes maps onto a documented feature of venous wound pathobiology, and the specific therapeutic targets they address are examined in the sections that follow.

### 2.5. Formulation Formats

The same polymer chemistry may be presented as an amorphous gel, a preformed sheet, a hydrogel-impregnated gauze or foam, a sprayable formulation or an in situ-forming injectable [57], and format determines clinical fit at least as much as chemistry does. Amorphous gels and impregnated gauzes act as primary contact layers and therefore require a secondary retention dressing, and they conform to irregular and undermined wound beds at the cost of a tendency to migrate under sustained compression. Sheets offer handling convenience and mechanical integrity but conform poorly to the malleolar contours where venous ulcers typically arise. Impregnated composites represent the most common commercially available compromise.

A consideration specific to this setting, and one that appears not to have been systematically addressed in the dressing literature, is that format also determines pressure distribution. The principle is already accepted for the layers above: compression bandages are applied over subcompression padding precisely to spread load across bony prominences and prevent friction and pressure damage [58]. The same logic applies downward. Bulk added over the malleoli or the tibial crest redistributes the pressure profile that the compression system is designed to deliver, so a thick sheet or a heavily loaded composite may locally compromise the therapy beneath which it is applied. In situ-forming systems remain largely investigational in this indication.

## 3. Mechanistic Rationale in the Venous Wound Microenvironment

### 3.1. Pathophysiological Targets Specific to Venous Disease

The case for hydrogels in venous disease is strongest when it is made against the specific pathobiology of the venous wound rather than against a generic model of impaired healing. That pathobiology is comparatively well described.

The proximate driver is ambulatory venous hypertension, arising from valvular incompetence, venous obstruction, or both, and transmitted to the dermal microcirculation [1,59]. Sustained microvascular hypertension produces capillary elongation and increased permeability, with extravasation of macromolecules and of erythrocytes into the interstitium. What follows from that extravasation has been explained in more than one way. Earlier accounts emphasized the pericapillary fibrin cuff as a barrier to oxygen and nutrient diffusion, and subsequent work implicated leukocyte trapping within the congested microcirculation and the sequestration of growth factors by extravasated macromolecules [60]. These proposals are not mutually exclusive, and each retains explanatory value, but the iron hypothesis has attracted the most attention in recent years because it identifies a chemically tractable target.

Erythrocyte breakdown releases hemoglobin, and the resulting deposition of hemosiderin produces the characteristic hyperpigmentation of the gaiter area [61]. This is not a cosmetic epiphenomenon. Systematic staining of skin biopsies across CEAP stages shows no hemosiderin in the normal skin of C2 to C4A limbs, whereas lipodermatosclerosis and ulceration are invariably accompanied by iron deposition, so that severe skin change does not occur until iron overload does [61]. Iron deposits generate reactive oxygen species (ROS) through Fenton chemistry, and the resulting oxidative environment sustains inflammation, drives excessive expression and activation of matrix metalloproteinases (MMP), promotes lipid peroxidation and contributes directly to ulceration [61,62]. Comparative measurements in limbs affected by severe chronic venous disease have demonstrated concurrent elevation of local iron indices and of MMP-9 relative to unaffected limbs and to controls [62]. Why the same deposits ulcerate some limbs and not others appears, in part, genetically determined, since the C282Y mutation of the HFE gene raises ulcer risk more than sixfold in primary chronic venous disease [63].

Proteolytic imbalance is a defining biochemical feature. Chronic wound fluid from venous ulcers contains markedly elevated gelatinases and collagenases, together with reduced tissue inhibitors of metalloproteinases (TIMPs), producing a net catabolic milieu in which newly deposited matrix, and indeed exogenously applied growth factors, are degraded before they can act [64,65]. Any bioactive protein delivered to such a wound without protection has a short functional half-life, which bears directly on how a delivery system must be designed.

**Figure 2:**
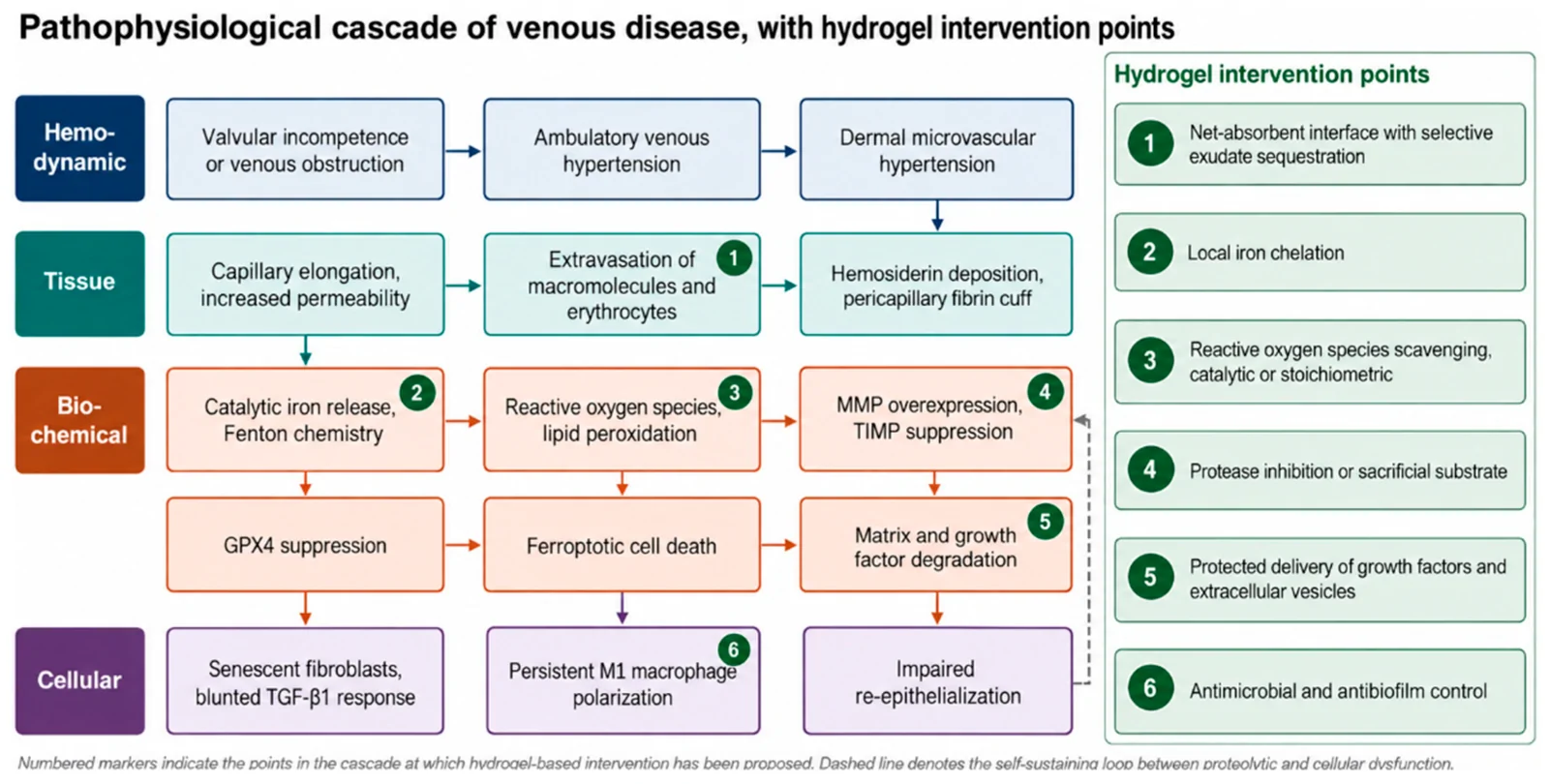
Pathophysiological cascade of venous disease and the points at which hydrogel-based intervention has been proposed. Ambulatory venous hypertension arising from valvular incompetence, venous obstruction, or both, is transmitted to the dermal microcirculation, where capillary elongation and increased permeability permit extravasation of macromolecules and erythrocytes. Erythrocyte breakdown yields hemosiderin deposits, and the pericapillary fibrin cuff forms in parallel. Catalytic iron released from these deposits drives Fenton chemistry, generating reactive oxygen species and lipid peroxidation, which in turn sustain overexpression and activation of matrix metalloproteinases with concurrent suppression of their tissue inhibitors. Lipid peroxidation coupled to progressive suppression of glutathione peroxidase 4 provides the substrate for ferroptotic cell death, and the resulting proteolytic environment degrades newly deposited matrix together with endogenous and exogenously applied growth factors. At the cellular level, fibroblasts acquire a senescent phenotype with blunted responsiveness to transforming growth factor beta 1 while remaining synthetically active, macrophages remain polarized toward a persistent pro-inflammatory state, and re-epithelialization is impaired. The dashed line indicates the self-sustaining loop by which cellular dysfunction feeds back onto proteolytic excess. Numbered markers correspond to the intervention points listed on the right: (1) a net-absorbent interface with selective sequestration of exudate constituents; (2) local iron chelation; (3) scavenging of reactive oxygen species, whether catalytic or stoichiometric; (4) protease inhibition or competitive absorption by a sacrificial substrate; (5) protected delivery of growth factors and extracellular vesicles; and (6) antimicrobial and antibiofilm control. MMP, matrix metalloproteinase; TIMP, tissue inhibitor of metalloproteinases; GPX4, glutathione peroxidase 4; TGF-β1, transforming growth factor beta 1.

The cellular compartment is equally altered. Fibroblasts cultured from venous ulcers display features of senescence, with impaired proliferative capacity and blunted responsiveness to transforming growth factor beta 1 (TGF-β1) that worsens with increasing clinical severity and with ulcer duration, while remaining synthetically active and thus continuing to contribute to a dysregulated matrix [66,67,68]. Macrophage polarization is skewed toward a persistent pro-inflammatory phenotype, a shift for which local iron loading has been implicated as a driver [69]. More recently, ferroptosis has been proposed as a mechanistic link between iron accumulation and tissue loss, with evidence of stage-dependent changes in cutaneous iron content and glutathione peroxidase 4 (GPX4) expression across the progression from hyperpigmentation through lipodermatosclerosis to frank ulceration [70]. This line of work remains preliminary but is conceptually important, since it identifies a druggable axis specific to venous rather than diabetic or pressure-related ulceration. Figure 2 presents these mechanisms schematically, with hydrogel intervention points superimposed.

### 3.2. Localized Delivery and Its Limits in Fibrotic Tissue

The delivery argument for hydrogels rests on two propositions: that local concentrations at the target tissue can be raised well above those achievable systemically, and that systemic exposure and its attendant toxicity can be minimized. Both are well established in principle, and the release profile can be tuned across a wide range through the network chemistries described above [54]. What is less often examined is whether the target tissue in advanced venous disease is actually reachable.

Venous disease offers a clinical precedent for this reasoning, though a more equivocal one than is usually acknowledged. Topical heparin preparations have been used for decades in superficial venous disorders, precisely because systemic anticoagulation carries bleeding risk disproportionate to the local inflammatory problem being treated [71,72]. What that history establishes is that a topical route has been accepted in venous pathology by clinicians and regulators alike, and that patients report symptomatic benefit. What it does not establish is efficient transport. Distribution studies of a 1000 IU/g gel place 45% of the applied dose in the stratum corneum, 11% in the deep epidermis and under 3% in the dermis, with dermal bioavailability of roughly 0.7% and microvascular availability below 0.1%, while about half the active substance never leaves the skin surface [73]. For a large, highly charged glycosaminoglycan, this is unsurprising, and it means that decades of accepted use rest on delivering a fraction of a percent of the dose to the compartment where the target lies. The precedent is therefore better read as a statement of clinical appetite, and of how much symptomatic benefit can apparently be obtained from very little delivered drug, than as proof of pharmacokinetic feasibility.

Two constraints specific to this tissue and this population are rarely considered in materials design. The first is access: the distribution figures above were measured on intact skin, and advanced disease adds dermal fibrosis, induration and impaired perfusion, so any strategy premised on reaching the periwound dermis rather than the ulcer surface must contend with a diffusion barrier that has not, to our knowledge, been characterized. The second is formulation freedom. Patch testing series report at least one positive reaction in 68 to 80% of venous ulcer patients, driven by excipients rather than actives, with sensitization increasing in proportion to ulcer duration [74,75]. Hydrogels are not exempt: positive tests to a marketed amorphous hydrogel occurred in 9.5% of one series [75]. Excipient screening therefore belongs in the development pathway, not in post-marketing surveillance.

### 3.3. Exudate, Oxidative Stress and Proteolysis as a Single Target Set

Moist wound healing, established as a principle more than half a century ago, remains the most frequently invoked justification for hydrogel use, and it is not wrong: a hydrated interface supports keratinocyte migration, permits endogenous proteolytic debridement and reduces pain at dressing change [76]. What the principle omits is that, in the venous ulcer, exudate is not merely fluid to be balanced. It is a biologically active medium carrying the proteases, iron species and inflammatory mediators described above, so retaining it in prolonged contact with the wound bed and the periwound skin is not self-evidently beneficial. Exudate management is, according to this view, a mechanistic intervention rather than a comfort measure, and the three targets conventionally treated as separate, namely, fluid, oxidative stress and proteolysis, are better understood as one circuit that a single material might interrupt at several points.

Antioxidant capacity is the most direct of those points. Polyphenol-containing networks, including tannic acid-crosslinked and polydopamine-functionalized systems, provide radical scavenging alongside adhesive and antibacterial properties [77]. Nanozyme incorporation, particularly cerium oxide and manganese-based systems, offers catalytic and therefore non-stoichiometric activity with the potential for sustained effect, though tissue retention and the toxicological profile of metal oxide particles under prolonged application to a wound with a compromised barrier remain incompletely characterized. Direct incorporation of enzymatic or small-molecule antioxidants is simpler and shorter-lived [78].

Proteolytic excess has been approached both through sacrificial substrates that competitively absorb activity and through incorporation of specific inhibitors. Collagen and oxidized regenerated cellulose composites are the clinically available embodiment of the first approach, and they act on serine proteases as well as metalloproteinases, binding and inactivating elastase, plasmin and gelatinases in venous wound exudate [79]. They carry more trial evidence than anything else in this category, though the evidence is itself equivocal: the pivotal diabetic foot trial found no significant difference in complete healing, with borderline benefit confined to ulcers of less than six months’ duration [80], and the venous trials have been small or have failed to reach significance [81]. These are lyophilized matrices rather than hydrogels, and the comparison should be made with that difference in view.

Iron chelation deserves separate emphasis. If local iron overload drives the oxidative and proteolytic injury of venous ulceration, a matrix functionalized with deferoxamine addresses this etiology at its source. Chelator-loaded hydrogels have attracted considerable attention in diabetic wounds, but for a different reason: deprived of Fe^2+^, the prolyl hydroxylases fail to mark HIF-1α for degradation, and the angiogenic response follows [82,83]. The venous indication, where a genuine local excess of catalytic iron is present and chelation would target it directly, has received comparatively little attention. We regard this as one of the clearest unexploited opportunities in the field. The counterargument follows from the same enzymology, since collagen prolyl hydroxylase is also iron-dependent, so the therapeutic window and spatial confinement of the chelator are the central questions any such system must answer.

Those questions deserve a direct answer, because the rationale stands or falls on them. Two families of iron-dependent dioxygenase are at stake, and they pull in opposite directions. Inhibition of the HIF prolyl hydroxylases is the desired effect, since it stabilizes HIF-1α and drives the angiogenic response that motivates chelator-loaded hydrogels in diabetic wounds [82,83]. Inhibition of collagen prolyl-4-hydroxylase is the undesired one, since it impairs triple helix formation and therefore matrix deposition. Both enzymes use Fe(II) and 2-oxoglutarate, so they cannot be separated by chelator chemistry, and the discrimination has to be spatial and kinetic instead. Here, the pharmacology is unexpectedly favorable. Deferoxamine is a large, hexadentate, highly hydrophilic siderophore with poor membrane permeability, and at therapeutic concentrations its action is predominantly on the extracellular labile pool, whereas deferiprone and deferasirox enter cells rapidly and chelate intracellular iron directly [84]. The chelator best suited to this indication is therefore the one already least able to reach the intracellular compartment. Covalent tethering to the network extends that separation: an immobilized chelator cannot be internalized at all; iron must diffuse to it rather than the reverse; and the ferrioxamine complex is physically removed when the dressing is changed rather than redistributed within the limb. The separation is nonetheless quantitative rather than absolute, since depleting extracellular labile iron establishes a gradient that promotes cellular efflux over time, so dose and application duration remain part of the answer rather than being solved by immobilization alone. What this yields is a falsifiable claim rather than an assumption, and the stage-one study proposed in Section 7.7 is designed to test it: extracellular labile iron in exudate should fall while intracellular labile iron in co-cultured keratinocytes does not HIF-1α and its downstream targets should be preserved or increased; and hydroxyproline deposition and keratinocyte migration should be unchanged (Supplementary Table S1). If a construct cannot demonstrate that separation, the etiological rationale for chelation in this indication does not survive, and the honest response is to abandon it rather than to report angiogenic endpoints and omit collagen ones.

### 3.4. Microbial Burden and Biofilm

Bacterial colonization is near universal in venous ulceration, and the distinction conventionally drawn is between colonization, which is compatible with healing, and the established biofilm, which is not. Biofilm confers tolerance to antiseptics and to systemic antibiotics far above planktonic minimum inhibitory concentrations and is implicated in up to 60% of chronic wounds [85]. This is also the most heavily populated corner of the hydrogel literature, with silver, iodine, quaternary ammonium compounds, antimicrobial peptides and phage preparations all delivered from hydrogel matrices [30].

Two findings temper the enthusiasm, and both are venous-specific. The first is the silver precedent noted in Section 2.2: a mechanistic rationale at least as persuasive as those now offered for peptides, which nonetheless showed no healing benefit under randomized evaluation [8]. The second is more fundamental. In a longitudinal study of 117 patients with venous ulcers under standardized weekly sharp debridement, wounds shrank in 96 of 117 cases, and the investigators concluded that biofilm may have little influence on healing under that regimen [86,87]. If the premise is weaker than assumed once debridement and compression are performed properly, then improving antimicrobial delivery may address a problem that is not rate-limiting.

### 3.5. Angiogenesis, Re-Epithelialization and Matrix Remodeling

The final mechanistic axis concerns active promotion of repair rather than removal of inhibition. Hydrogel systems have been used to deliver vascular endothelial growth factor and other angiogenic factors, platelet-derived preparations, mesenchymal stromal cells and, increasingly, cell-free extracellular vesicles. The matrix serves two functions here: it localizes the payload at the wound site and protects a labile agent from rapid clearance and from the proteolytic environment described above, which is the principal obstacle to translating vesicle therapy in particular [88,89]. Conductive hydrogels incorporating polypyrrole, polyaniline or carbon-based fillers rest on a firmer rationale than is often assumed, since endogenous electric fields of 40 to 200 mV/mm direct keratinocyte migration, fibroblast proliferation and angiogenesis after injury, and these fields are disrupted in chronic wounds [90]. Evidence in venous ulceration specifically is nonetheless absent, with the published systems having been tested in diabetic or infected wound models [91]. Decellularized matrix preparations occupy an intermediate position, supplying architectural and biochemical cues without a defined single agent, although the products available clinically in this class are acellular matrices rather than hydrogels [92].

A general caution applies across this category. Each of these payloads is biologically plausible, each has generated encouraging rodent data, and none has yet demonstrated reproducible benefit in venous ulceration in adequately powered clinical trials. The reasons are examined below.

## 4. Therapeutic Applications Across the Venous Disease Spectrum

### 4.1. Compression as the Therapy and the Dressing as the Adjunct

Compression is the therapy and the dressing is the adjunct. Every claim made for a hydrogel in venous ulceration is therefore a claim about incremental benefit added to adequate compression, and any trial that does not standardize compression across arms is uninterpretable regardless of what its dressing arms contain.

Three interactions follow from that relationship and shape what a hydrogel in this setting must tolerate. Mechanically, a gel held under sustained interface pressure of 30 to 40 mmHg is subject to creep, migration and expression from beneath the bandage, particularly in the malleolar hollows where conformability is most needed, and this is a formulation problem that rheological characterization under representative loading would identify early. Pharmacokinetically, sustained compression alters the release profile of a loaded system through its effects on swelling equilibrium and convective transport, an interaction that appears not to have been reported in the wound hydrogel literature and that would be straightforward to model. Clinically, the dominant practical variable is wear time, since bandage change intervals govern nursing cost, and a dressing that fails before the bandage does forfeits the economic argument entirely.

### 4.2. Hydrogel Dressings for Venous Leg Ulceration

Hydrogel dressings entered venous ulcer care through the debridement route rather than through any regenerative claim. Amorphous gels donate moisture to devitalized tissue, soften slough and necrotic eschar, and thereby support autolytic debridement without the pain and the poor tissue selectivity of sharp or mechanical methods. This remains their clearest and most defensible indication, and it is reflected in guideline language that positions hydrogels as one option among several rather than as a preferred primary dressing [15,93].

One practical caution belongs here rather than later, because the sloughy ulcer of uncertain etiology is precisely the wound to which an amorphous gel is most likely to be applied reflexively. No ulcer should be dressed and compressed before the ankle-brachial pressure index has excluded significant arterial disease, since compression on an ischemic limb can precipitate tissue necrosis. The hydrogel contributes to that hazard in two secondary ways: a moisture-donating material on a poorly perfused limb favors maceration, and the appearance of active management can delay appropriate vascular referral [58].

Commercially available products span amorphous gels, preformed sheet dressings with or without an adhesive border, hydrogel-impregnated gauzes, and composite constructs in which a hydrogel contact layer is bonded to an absorbent foam or superabsorbent core. These formats do not share an indication, and the distinction matters more here than in most wound types. The amorphous gel earns its place on the dry or sloughy wound described above, whereas the composite is designed to preserve a non-adherent, atraumatic interface while removing rather than donating fluid, which in principle suits the hyperexudative ulcer that constitutes most of the venous caseload [57].

Positioning relative to alternatives therefore follows from fluid-handling capacity more than from any other property. Alginates and hydrofibers are conventionally selected for moderate to heavy exudate, foams handle high volumes and distribute pressure, hydrocolloids support autolysis in low-to-moderate exudate, and classical hydrogels sit at the dry or sloughy end of this spectrum [94]. A substantial proportion of venous ulcers does not fall at that end, which narrows the population for which a classical hydrogel is the appropriate choice and may partly explain the flat comparative results that controlled evaluation has produced [95].

Where these materials might be expected to perform distinctively well is in pain at dressing change and in patient acceptability, since a hydrated, non-adherent interface lifts without disturbing the wound bed. Whether they in fact do so is unknown: no trial in the Cochrane synthesis reported pain, quality of life or any patient-reported outcome [13]. The picture is complicated further by the sensitization data discussed above, since hydrogel products appear among the agents to which this population reacts on patch testing. For a condition whose burden is measured in years of discomfort, odor and social restriction, the one advantage most plausibly attributable to this class of material is also the one that has never been tested [75].

### 4.3. Antimicrobial-Loaded Systems and Biofilm Control

Given the tolerance that biofilm confers and the persistent inflammatory stimulus it sustains, antimicrobial delivery from a hydrogel matrix has an obvious rationale, and hydrogels have been used as vehicles for essentially every antimicrobial class relevant to wound care. Ionic and nanoparticulate silver remains the most widely commercialized, followed by cadexomer and povidone iodine formulations, medical-grade honey, chlorhexidine and polyhexanide. Investigational approaches include antimicrobial peptide-conjugated networks, quaternized chitosan derivatives with intrinsic membrane-disrupting activity, bacteriophage-loaded gels, photothermal and photodynamic systems using near-infrared-responsive fillers, and nitric oxide-releasing matrices [96].

Two of these classes face obstacles specific to the venous setting that are rarely acknowledged in the papers proposing them. Antimicrobial peptides are substrates for the same gelatinases and collagenases that are elevated in venous wound fluid, so a peptide delivered unprotected into that environment has a functional lifetime measured against a proteolytic burden rather than against its own release kinetics. Photothermal and photodynamic systems require light delivery to the wound surface, which a multilayer compression bandage worn for up to 7 days precludes between changes, and the resulting treatment schedule bears little resemblance to the one used in preclinical models [97].

The clinical evidence for this category is instructive and largely negative. The most direct test remains a randomized trial of silver-donating dressings beneath compression in 213 patients with venous ulcers, which found no difference in healing at 12 weeks, 59.6% against 56.7%, no difference in any secondary endpoint, and a significantly higher cost [8]. Cochrane synthesis reached the same conclusion across agents, with no between-group differences in complete healing for honey-based products against usual care, for silver sulfadiazine ointment against standard care, or for silver-impregnated dressings against non-antimicrobial dressings [98]. A separate review of topical silver in infected chronic wounds found the available trials too few and too short to support any recommendation [99]. A network meta-analysis did detect a signal favoring silver over non-adherent dressings, though the network as a whole carried low certainty and the authors concluded it could not even identify which comparison to prioritize next [15].

The reasonable clinical position is that antimicrobial hydrogels have a role in wounds with clinical signs of local infection or suspected biofilm burden, applied for a defined period with review, rather than as routine prophylaxis. The reasonable research position is that biofilm disruption, rather than bacterial killing in planktonic culture, should be the primary in vitro endpoint for any new antimicrobial hydrogel intended for this indication.

### 4.4. Bioactive and Regenerative Payloads

The regenerative rationale is that a venous ulcer represents not merely an unhealed wound but an arrested one, in which the cellular and signaling machinery of repair has been disabled by the mechanisms described above. Supplying the missing signal, or the missing cell, is therefore an attractive proposition, and the hydrogel matrix serves two distinct functions: spatial retention at the wound site and protection of a labile payload from a proteolytic environment that would otherwise degrade it within hours.

Four payload families have been pursued along this line, and Table 2 sets out the level of evidence each one has reached. Growth factor delivery has the longest history, with recombinant platelet-derived growth factor approved in diabetic foot ulceration [54]. The disappointing translation to venous ulceration is usually attributed to the protease burden, inadequate residence time, and the improbability that a single factor can reconstitute a multistep program. Encapsulation, heparin-mimetic affinity binding and covalent tethering have all been used to extend functional half-life [54]. Platelet-rich plasma occupies an intermediate position between growth factor and cell therapy. It is autologous and therefore regulated variably across jurisdictions. The literature is methodologically heterogeneous, yet it carries the largest effect estimate of any intervention discussed in this review: meta-analysis of 16 randomized trials reports an odds ratio of 5.06 for complete healing and a reduction of 3.25 months in time to closure [100,101]. The fact that such a crude preparation outperforms every engineered system in the venous evidence base warrants deliberate reflection and suggests that payload sophistication has been a poorer predictor of clinical benefit than the simple delivery of a broad, autologous, protease-resistant mixture to the wound surface. Cell-based approaches range from encapsulated mesenchymal stromal cells and adipose-derived stromal vascular fraction to bilayered living skin equivalents. The latter represent the clinically translated end of this line, with demonstrated benefit in venous ulceration at a cost that has limited adoption; these are cell-seeded collagen constructs rather than hydrogels and the comparison should be made with that difference in view [102]. Cell-free extracellular vesicle therapy has largely displaced whole-cell delivery in recent preclinical work, on the grounds that the stromal cell effect is predominantly paracrine, and EV-loaded hydrogels have produced encouraging rodent results for angiogenesis, macrophage repolarization and re-epithelialization [88], though no clinical trial in venous ulceration has yet reported.

### 4.5. Symptom-Directed and Venoactive Payloads

A different logic governs the payloads aimed at symptoms rather than at closure, and it deserves more attention than it usually receives, since patients consistently rank pain above healing rate among the outcomes that matter to them. Ibuprofen-releasing foam dressings are the principal commercial embodiment of that logic and provide a template that hydrogel systems could adopt, a locally delivered non-steroidal agent achieving symptomatic benefit without meaningful systemic exposure, although the marketed products are polyurethane foams rather than hydrogels. Topical corticosteroids have a place in stasis dermatitis of the periulcer skin, applied to that skin rather than to the ulcer bed, where the balance between inflammatory control and impaired epithelialization requires care.

Antioxidant and iron-directed payloads are, on mechanistic grounds, the most specific opportunity available in this indication, and the relevant question is one of development stage rather than of rationale. Polyphenolic networks, nanozyme composites and enzymatic antioxidants have all reached proof of concept in animal models [20,103], and some agents span both targets, since alpha-lipoic acid scavenges radicals directly while also chelating the iron and copper that catalyze their formation [103]. Since excess reactive oxygen species drive MMP overexpression, reducing the oxidative burden should also lower proteolytic activity, addressing two coupled abnormalities with a single material, although published systems have used MMP activity as a release trigger rather than demonstrating its suppression [104]. None has entered controlled evaluation in venous ulceration, and iron-chelating hydrogels remain conspicuously underexplored despite the strength of the mechanistic case.

The venoactive compounds constitute the most speculative subcategory and, for a materials readership, potentially the most interesting. Flavonoid preparations, horse chestnut seed extract, Ruscus extracts and calcium dobesilate all have established oral use in chronic venous disease, with effects attributed variously to reduced capillary permeability, inhibition of leukocyte adhesion, modulation of venous tone and enhanced lymphatic drainage [105]. Calcium dobesilate illustrates the argument for a route that limits systemic exposure, since a case-control study found recent exposure in 5.6% of agranulocytosis cases against 0.4% of controls, with two positive rechallenges [106], although a subsequent pharmacovigilance review placed the prevalence at 0.32 cases per million treated patients and attributed the signal to methodological bias [107]. Whichever reading is correct, a systemic hazard that is disputed rather than excluded is precisely the situation in which local delivery has most to offer. Pentoxifylline occupies a distinct position, since meta-analysis of 10 studies and 1025 participants reports healing in 62% of patients at the 1200 mg dose, making it one of the few pharmacological adjuncts with reproducible benefit in this condition [108,109,110].

The translational question that follows is whether locoregional hydrogel delivery to the gaiter area could achieve tissue concentrations unattainable by the oral route while avoiding the gastrointestinal intolerance that limits adherence to systemic therapy. Two obstacles stand in the way: several of these compounds are poorly water soluble, which points toward micellar, cyclodextrin-complexed or nanoemulsion-loaded architectures, and several are large enough that transport across the stratum corneum, and then through indurated dermis, is the limiting step rather than the formulation. As far as we are aware, no controlled clinical data support topical delivery of any venoactive compound in this indication as far, and this passage should be read as an argument for investigation rather than a summary of established practice.

### 4.6. Hydrogels Beyond the Ulcer

The absence of controlled data for topical venoactives in the ulcer is all the more striking given what surrounds it. Attention to venous ulceration has tended to obscure the fact that gel formulations have been in continuous clinical use for other manifestations of venous disease for several decades, and that this experience carries lessons for the ulcer indication.

Topical heparin and heparinoid preparations are used in SVT, post-sclerotherapy inflammation, superficial phlebitis of infusional origin and symptomatic varicose disease. Heparin possesses anti-inflammatory as well as anticoagulant activity, and topical administration permits exploitation of the former without the systemic bleeding risk associated with the latter. Reported clinical experience includes improvement in induration, pain, swelling and limb function with a heparin gel at 1000 IU/g applied over 4 weeks in superficial thrombophlebitis, alongside a substantial body of older phase II to IV data [71,111,112]. Topical non-steroidal anti-inflammatory drugs have similarly shown superiority over placebo for local symptom relief in infusional thrombophlebitis [110].

Two observations follow. The first is encouraging: a gel vehicle has sustained decades of clinical use on the basis of symptomatic benefit, which establishes clinical and regulatory acceptability for a topical route in venous pathology, even though, as the distribution data in Section 3.2 show, it establishes nothing about transport efficiency. The second mirrors the ulcer literature exactly, in that published research on the efficacy and safety of these preparations is scarce, much of the supporting evidence is uncontrolled or dated, and formulation variables such as heparin concentration and the presence of permeation enhancers are known to influence outcome without having been systematically optimized [111].

The engineering required to address this is not speculative. Modern network chemistries offer controlled and sustained release rather than bolus application [54], adhesive formats that maintain conformal contact over a phlebitic segment through a full dosing interval [45], and microneedle architectures capable of delivering high-molecular-weight actives past the stratum corneum. A thrombin-responsive microneedle patch that senses activated thrombin and releases heparin in a feedback-controlled manner has already been built, though it was directed at systemic anticoagulation rather than at local control of a superficial thrombotic segment [113]. Redirecting that architecture toward superficial venous thrombosis is a concrete near-term target rather than a further iteration of the wound dressing, and one with a shorter path to evaluation than any chronic ulcer indication, since the endpoints are symptomatic and measurable within weeks, and an accepted comparator already exists.

## 5. Preclinical and Clinical Evidence: A Critical Appraisal

### 5.1. In Vitro Evidence

The characterization package accompanying a new wound hydrogel has become highly standardized: swelling ratio and degradation profile, compressive or rheological measurement, scanning electron microscopy of the porous architecture, in vitro release of the loaded agent, antibacterial testing against reference strains of *Staphylococcus aureus* and *Pseudomonas aeruginosa*, cytocompatibility by a metabolic assay such as MTT or CCK-8 on a fibroblast or keratinocyte line, and a scratch closure assay presented as evidence of migration promotion.

This package is reproducible and comparable across laboratories, which is a genuine strength, but its predictive relationship to clinical healing is weak and it is worth stating why. Scratch closure in a confluent monolayer of immortalized cells measures a process only distantly related to re-epithelialization across a chronic wound bed. Antibacterial testing against planktonic reference strains does not predict activity against a mature polymicrobial biofilm, and agar diffusion methods, in particular, are poorly suited to materials whose active component does not diffuse. Cytocompatibility assessed in healthy fibroblasts says little about behavior in the presence of the senescent, TGF-β1-unresponsive population that characterizes venous ulceration [59,114,115], and metabolic assays are themselves prone to artifacts in the presence of redox-active materials, which are precisely the class most often proposed for this indication. Release kinetics measured in phosphate-buffered saline bear an uncertain relationship to release into a protease-rich, protein-laden exudate.

Most consequentially, no in vitro system reproduces ambulatory venous hypertension. Models incorporating cyclic loading at physiological pressures, iron loading of the culture environment, or wound fluid drawn from patients with venous leg ulcers would be more informative than any of the standard assays, and their absence means that the venous specificity claimed by many published systems has never actually been tested.

### 5.2. Animal Models and Their Translational Limitations

Rodent skin differs from human skin in ways that matter here. The presence of the panniculus carnosus permits a degree of contraction that human skin does not, and although the extent of that difference has been overstated, with re-epithelialization and contraction each accounting for roughly 40 to 60% of initial closure in murine excisional wounds [116], splinting is now routinely applied precisely because unrestricted contraction confounds quantitative evaluation [117,118]. The more consequential mismatch is biological rather than mechanical. An acute excisional wound in a healthy young animal is the opposite of a chronic wound in an elderly patient, and a systematic review of 129 preclinical wound studies makes the point directly: modeling a disease of aging in eight-week-old mice cannot capture the fibroblast senescence and stem cell exhaustion that define the clinical problem [119]. Absent too are the protease excess, the biofilm and the sustained inflammatory drive. Above all, no animal model of venous ulceration has been validated against the human pathobiology or brought into routine use; preparations based on venous ligation, arteriovenous fistula creation or iron injection each reproduce individual features, but none recapitulates the sequence from ambulatory venous hypertension through lipodermatosclerosis to ulceration.

### 5.3. A Minimal Preclinical Battery for Venous-Specific Claims

The criticisms above are only useful if they come with a replacement. We therefore set out the minimum a preclinical package would need to contain before a hydrogel could be described as designed for venous ulceration rather than for chronic wounds in general. Supplementary Table S1 gives the full battery with conditions and acceptance criteria; three components carry most of the weight.

The first is mechanical. A material for this indication should be fatigued, not merely characterized: hydrated, at 37 °C, in simulated wound fluid, through approximately 5 × 10^4^ compressive cycles between 5.3 and 10.7 kPa at 1 Hz, with storage modulus, thickness, mass and net fluid uptake measured before and after (Section 7.5). A gel that has only been tested at rest has not been tested for the environment it will occupy.

The second is proteolytic. Degradation and release should be measured in pooled human venous ulcer exudate rather than in buffer, at a fluid-to-gel ratio matched to the exudate output of the target wound class, over a full bandage interval. The pool should be characterized and reported, minimally for MMP-9 by zymography and immunoassay, the MMP-9 to TIMP-1 ratio, total protein and pH, since these vary widely between donors and a result reported against an uncharacterized pool cannot be reproduced elsewhere. One control distinguishes this experiment from a descriptive one: a parallel arm in exudate treated with a broad-spectrum metalloproteinase inhibitor, which separates proteolytic degradation from simple hydrolysis. For systems claiming protease-responsive release, the release rate must be shown to scale across at least three levels of MMP-9 activity; a system that releases at the same rate whatever the protease burden is not responsive, whatever its chemistry [62,120].

The third is microbiological. Antimicrobial claims should be tested against a mature dual-species biofilm of *Staphylococcus aureus* and *Pseudomonas aeruginosa*, established for at least 48 h before treatment, grown under continuous low shear in a drip flow reactor under continuous low shear [121] with simulated wound fluid or pooled exudate as the medium rather than a rich laboratory broth, and challenged by a single application held for the intended wear time rather than by repeated dosing. Clinical isolates should be included alongside reference strains. The conventional bar of a 3 log_10_ reduction against an untreated control is a reasonable minimum, and a vehicle-only comparator is essential, since a hydrogel that physically occludes and hydrates a wound bed will alter bioburden without any antimicrobial payload. We would add a constraint that follows from Section 3.4: given the evidence that biofilm may not be rate-limiting once debridement and compression are delivered properly, results from this assay support a bioburden claim and not a healing claim [85,86,87].

Two further points apply across the battery. Cytocompatibility should be assessed on senescent or stress-induced human dermal fibroblasts with confirmed loss of TGF-β1 responsiveness, since healthy young lines cannot model the cell population that defines this disease, and metabolic assays such as MTT and CCK-8 should be paired with an orthogonal non-redox readout whenever the material is redox-active [122], which describes most of what is currently proposed here. Where antioxidant or chelation activity is claimed, labile iron should be measured directly in the test medium [123], and the delivered chelator dose should be shown not to inhibit collagen prolyl hydroxylase, which is itself iron-dependent (Section 3.3) [59,114,115].

None of this requires apparatus that a materials laboratory does not already own. What it requires is access to human venous ulcer exudate, which is the one input a materials group cannot generate alone, and which is the practical reason this battery has not been adopted. That is an argument for collaboration with a wound care service rather than for a different assay.

### 5.4. Clinical Trial Evidence

The most directly relevant synthesis is the Cochrane review of hydrogel dressings for VLU, which searched to May 2021 and included four randomized controlled trials randomizing 272 participants in total against gauze and saline, alginate, manuka honey and hydrocolloid dressings; trials of hydrogels impregnated with antimicrobial, antiseptic or analgesic agents were excluded by protocol, having been addressed in separate reviews [14]. Two features of that population deserve comment. Women constituted a minority of the reported participants, although the condition has a female preponderance, so the trial population does not represent the population that will receive the intervention. And the review concluded that it cannot be determined whether hydrogel dressings are more effective than these alternatives, with certainty very low across every comparison, downgraded for risk of bias and imprecision.

The absence of four outcomes is arguably more important than the null finding on healing. No included study provided data on ulcer recurrence, health-related quality of life, pain or cost [14], which is to say that the evidence base is silent on most of what matters to patients and to health systems in a condition defined by chronicity and recurrence. This omission is total rather than partial, and it recurs as a constraint throughout the remainder of this review.

The broader picture from the network meta-analysis of dressings and topical agents is consistent. A signal favoring silver dressings over non-adherent dressings was detected and assessed as moderate certainty for that specific comparison, but the authors concluded that confidence in any conclusion was limited because the network as a whole represented low-certainty evidence, and that the analysis was uninformative even with respect to which interventions should be prioritized in a future large trial [15]. After three decades of trials and more than 7000 randomized participants, the field cannot say which dressing to use, nor can it say which comparison to run next. Table 3 sets out these syntheses with their scope, findings and certainty.

An informative contrast comes from the diabetic foot. Pooling three trials, Cochrane synthesis found significantly greater healing with hydrogel than with basic wound contact dressings (RR 1.80, 95% CI 1.27 to 2.56) [124], although the pooled studies differed in follow-up duration and ulcer severity. A later meta-analysis spanning both etiologies graded this as moderate-quality evidence and found no difference for any other dressing comparison [95]. The asymmetry admits three readings, and the available data do not distinguish between them. The first is that hydrogels exert a real effect that becomes detectable in an etiology where the dominant wound problem is dry necrotic tissue requiring debridement, which is exactly what an amorphous gel is designed to address. The second is that the effect is real in both settings but masked in venous disease, where effective compression addresses the principal pathophysiological driver and any incremental contribution of the wound contact layer must be detected against that background, a situation in which underpowered trials will systematically miss a small true effect. The third, and simplest, is that the intervention is matched to the mechanism in one condition and not in the other: in the diabetic foot, the primary dressing acts on the dominant local problem, whereas in venous ulceration the dominant problem is hemodynamic and no material applied to the wound surface reaches it.

### 5.5. Why the Evidence Gap Persists, and What Would Close It

It would be convenient to conclude that hydrogels do not work in venous ulceration. We conclude, instead, that the question has not been asked in a way capable of producing an answer, and that the reasons are structural rather than accidental.

Since compression dominates the treatment effect, the increment attributable to the dressing is small by construction, and trials powered for large effects will not detect it. Adequately powered comparative trials would require participant numbers an order of magnitude above those typically enrolled, which no current funder or sponsor has shown willingness to support. Compounding this, adherence to compression is frequently unreported, and an uncontrolled cointervention sitting directly on the causal path is more damaging than any imbalance in baseline characteristics.

Blinding presents a problem that sample size cannot solve. Dressings differ visibly and in their handling, participants and treating staff cannot be blinded, and healing is often assessed by unblinded personnel; thus, systematic bias affects every trial in this literature, regardless of its size. Outcome ascertainment is itself variable, since the ulcer area is measured by methods ranging from manual tracing to digital planimetry, with modest reproducibility between them.

Endpoint selection compounds these problems. Complete healing at 12 or 24 weeks dominates, which is a binary reduction of a continuous process and one that discards the information carried by the rate of area reduction; a substantial proportion of large ulcers will not close within that window, whatever is applied to them, which compresses the difference between arms. Set against the four unreported patient-centered outcomes noted above, this is a poor match between what is measured and what matters, and a core outcome set consistently applied would change the interpretability of this literature considerably.

Population heterogeneity generates further noise, since ulcer duration, area, depth, exudate level, bacterial burden and mixed arterial contribution all influence healing, and trials that do not stratify on the major prognostic variables cannot recover a modest treatment effect from the variance those variables introduce. The funding structure offers no correction: wound dressings are regulated as devices in most jurisdictions; market entry does not require efficacy trials of the scale expected for pharmaceuticals; and manufacturers therefore have limited commercial incentive to fund definitive comparative studies, while publicly funded trials in wound care remain rare relative to the disease burden.

Finally, the materials literature and the clinical literature have evolved separate reporting conventions, and the gap between them is now an obstacle in itself. Preclinical hydrogel papers seldom report the parameters a clinician would need to predict performance, such as fluid-handling capacity under load or expected wear time, while clinical trials seldom characterize the material tested beyond its trade name, which makes it impossible to determine whether two trials evaluated comparable products. Convergence on a minimum reporting standard for wound hydrogels, analogous to reporting guidelines adopted in other translational fields, would be a modest intervention with substantial returns and is the one recommendation in this review that requires no new funding to implement.

## 6. Translational and Regulatory Barriers

The physical constraints that a hydrogel must satisfy in this indication have been set out above: net absorption under sustained interface pressure, mechanical persistence and positional stability across the bandage change interval, adhesion that coexists with atraumatic removal from compromised periwound skin, and compatibility between the payload and terminal sterilization. Taken individually, each is a solvable materials problem. What follows concerns a different class of obstacle, one that acts after the material performs as intended in the laboratory and that accounts for much of the attrition between an elegant published system and a product that a district nurse can apply.

### 6.1. Manufacturing Scale-Up and Reproducibility

A considerable proportion of published systems involve multistep syntheses, custom monomers, nanomaterial components requiring their own characterization and quality control, or biological payloads with donor-dependent variability. Translating such a system to reproducible manufacture under good manufacturing practice (GMP), with defined critical quality attributes and acceptance criteria, is a distinct scientific and financial undertaking that is rarely addressed at the point of publication. Sterility adds a further constraint, since aseptic manufacture remains available as a fallback where terminal sterilization would damage the network or its payload, but at substantially greater cost and with a corresponding effect on the unit price that any economic argument must then defend.

### 6.2. Cost, Reimbursement and Health System Considerations

Advanced dressings carry higher unit costs than simple non-adherent alternatives, and formulary decisions are frequently made on that basis alone. This is analytically incorrect, since the purchase price of a dressing is only one component of the cost of an episode of care [125]. Linked routine data from Wales place the annual cost of district nurse visits for venous ulceration at £67.8 million, against £828,790 for dressings and compression bandages combined, with nurse visits identified as the primary cost driver and the direct cost per patient standing at £7706 per year [126]. Against a ratio of that magnitude, a dressing that safely extends the change interval or shortens healing time can lower total episode cost several times over despite a higher unit price.

The difficulty is that the analytical correction has no obvious addressee. Reimbursement for wound dressings is typically set per unit and administered separately from the community nursing budget that would absorb the saving, so the party paying the higher price is not the party benefiting from the reduced visit frequency. An economic case built on total episode cost therefore requires a payer able to see both sides of the ledger, and in many systems no such payer exists. Given that no trial in the Cochrane synthesis reported cost data at all [14], formal economic evaluation alongside a well-designed clinical trial, with wear time and nursing contacts recorded as outcomes in their own right, would be more informative in the short term than further material innovation.

The correction can be made quantitative, and doing so is more persuasive to a payer than the ratio alone. Let D be episode duration in days, *I* the interval between dressing changes, *c_v* the cost of a district nurse visit and *c_d* the cost of the dressing and bandage consumed at each change. Change-driven episode cost is then *C = (D/I)(c_v + c_d)*, and a material that extends the interval from *I*_0_ to *I*_1_ while multiplying its own unit cost by a factor *k* changes that cost in the ratio

C_1_/C_0_ = (I_0_/I_1_) × (c_v + k·c_d)/(c_v + c_d)

from which the break-even multiplier, the factor by which unit price may rise before the saving is exhausted, follows directly:

k* = [(I_1_/I_0_)(c_v + c_d) − c_v]/c_d

The Welsh figures supply the only input that is not device-specific. Taking one dressing set per visit, the ratio of £67.8 million in nurse visits to £828,790 in dressings and compression combined implies *c_v ≈ 82 c_d* [126]. On that basis, extending the change interval from daily to every third day reduces the change-driven episode cost by 67% at an unchanged unit price, by 65% even at a fivefold unit price increase, and reaches break-even only at roughly 167 times the current price. For the interval more typical of venous ulcer care under compression, three days to seven, the reduction is 57% and break-even falls at about 111 times.

Three caveats belong with these figures. The ratio is derived from aggregate national totals rather than reported directly; the calculation covers change-driven cost only and therefore overstates the effect on total cost of care, which includes hospitalization, debridement and the management of complications; and it assumes that healing time is unchanged. That last assumption is the one that matters. A longer interval that delays healing lengthens *D* and can cancel the saving entirely, which is precisely why wear time and healing must be measured in the same trial (Section 7.7) rather than argued separately. Set against these limitations, the conclusion is nonetheless robust to any plausible input: under a cost structure of this shape, unit price is very nearly irrelevant, and a development strategy optimizing for it is optimizing for the wrong variable.

### 6.3. Regulatory Classification and Its Consequences

Regulatory classification is determined by principal mode of action, and this has direct consequences for development strategy. In the European Union, a hydrogel acting through moisture management is a medical device under Regulation (EU) 2017/745, classified by Annex VIII Rule 4 for non-invasive devices in contact with injured skin. The class depends on the depth of the wound: a dressing intended principally for wounds that have breached the dermis and can heal only by secondary intention falls into Class IIb, above the simple non-adherent products it would replace, with a correspondingly heavier notified body conformity assessment [127]. Incorporation of a substance with action ancillary to that of the device engages Rule 14 and triggers consultation with the relevant medicines authority, while substance-based formulations that are absorbed or dispersed engage Rule 21. Both sit at a borderline where classification depends on objective evidence that the primary therapeutic effect is achieved by physical or physicochemical means [127,128]. Where the substance provides the principal action, the product is a medicinal product, with a corresponding change in the scale of evidence required, and products containing viable cells or tissue-engineered components fall under the advanced therapy medicinal product framework [129].

The same logic operates differently in the United States. A hydrogel making only moisture management claims is a Class II device cleared through 510(k), or through De Novo classification where no predicate exists. However, where the primary mode of action is chemical, the product becomes a combination product under 21 CFR 3.2(m) assigned to drug-led review, requiring an investigational new drug application, manufacturing controls, nonclinical toxicology and clinical efficacy data that a 510(k) does not demand. A chelating hydrogel can therefore be a Class III device in Europe under Annex VIII Rule 14 and a drug-led combination product in the United States on identical evidence, which is a further reason why classification is settled by design decisions taken long before any regulatory conversation begins.

Antimicrobial hydrogels sit at a boundary that has proved variable in practice, since in the European Union an antimicrobial claim may engage biocidal product legislation alongside the device framework, and the classification of silver-containing dressings, in particular, has been a source of protracted uncertainty. The position differs again in the United States, where a simple dressing may reach the market through the 510(k) pathway while an active-containing construct is handled as a combination product with a designated primary mode of action. Adding a bioactive payload to a hydrogel is therefore not an incremental modification but a decision that may shift the development program by an order of magnitude in cost and duration, and preclinical work would be better targeted if this were factored in at the design stage rather than encountered afterwards.

## 7. Future Directions

### 7.1. Stimuli-Responsive and Sensing Systems

Responsive hydrogels release payload in response to a feature of the local environment rather than on a fixed schedule, and the venous wound offers several usable triggers. The chronic non-healing wound is alkaline, with surface pH commonly above 7.5, and acidification accompanies the transition toward healing, so pH-triggered systems in this setting must be designed to respond to elevated rather than reduced pH, which is the opposite of the convention in tumor-targeted delivery [130]. Reactive oxygen species and excess protease activity are available as alternative triggers, and protease-responsive systems are the most conceptually attractive of the three, because the trigger is itself a therapeutic target: release scales with the abnormality being treated and falls as that abnormality resolves, which produces a self-limiting delivery profile without any external control. Temperature is the least promising trigger here, since the differential associated with infection is of the order of a few degrees and a multilayer compression bandage is itself an insulating layer that confounds it.

Sensing extends the concept from therapy to monitoring, and the venous indication imposes a constraint that the wound sensing literature rarely addresses. A colorimetric indicator in the wound contact layer is unreadable beneath three or four bandage layers, so the output must sit at the outer bandage surface or be transmitted electronically: a wicking channel to a colorimetric window at the proximal margin, which must remain patent under the 5.3 to 10.7 kPa the dressing experiences (Section 7.5), or a battery-free near-field communication tag read by a phone held against the bandage [131].

The case for this is economic rather than clinical. District nurse visits for venous ulceration in Wales cost £67.8 million annually against £828,790 for dressings and compression combined [126], so the bandage change interval, not the healing rate, is where the money in this disease sits. Wear time is currently recorded from nursing notes by recall, which is why no trial has reported it and why a dressing whose only benefit is that it lasts seven days instead of three would be economically transformative and clinically invisible. In the trial proposed in Section 7.7, sensor-derived wear time serves two roles that should not be conflated: an objective adherence log replacing nurse-reported compression receipt, and a resource-use outcome without which the payer argument in Section 6.2 cannot be made [132]. Surrogacy is a third and more speculative role; pH falls as a wound transitions toward healing [130], but the trial should test that trajectory against percentage area reduction at four weeks [133] rather than assume it.

### 7.2. Injectable, In Situ-Forming and Sprayable Systems

In situ-gelling formulations, whether thermoresponsive, ionically triggered or photocrosslinked, permit conformal filling of irregular and undermined wound beds and open up the possibility of perilesional rather than surface application. That possibility is worth stating carefully, because it addresses the diffusion problem described earlier: the pathology in advanced disease resides in the dermal compartment of an indurated, poorly perfused limb, and a material deposited on the ulcer surface may never reach it. Injection into lipodermatosclerotic skin is, however, painful and would require local anesthesia, which limits what can realistically be delivered in a community setting and points toward this being a specialist-administered intervention if it is pursued at all.

Sprayable formulations deserve more attention than they usually receive and may be the most realistic format innovation for this indication. They conform to irregular geometry, can be applied rapidly by nursing staff without instrumentation, add negligible bulk beneath the bandage and therefore do not distort the pressure profile, and lend themselves to repeated application at each bandage change. The engineering problem is achieving adequate residence time from a thin deposited layer, which is precisely where dynamic covalent and rapidly gelling chemistries are relevant, and where the thixotropic recovery and high shear viscosity targets in Section 7.5 become the design specification rather than a general aspiration.

### 7.3. Bioprinting and Patient-Specific Constructs

Extrusion and light-based bioprinting allow for spatially graded constructs with distinct compositions at the wound margin and center, and in principle permit geometry matched to an individual ulcer, which is an attractive proposition for a wound whose margin and base differ biologically. The practical barriers are substantial. No care pathway currently supports point-of-care manufacture for a condition managed predominantly in community clinics and patients’ homes, and the regulatory status of a patient-specific combination product remains unsettled. The realistic near-term contribution of this technology to venous disease is therefore as a research tool, for constructing the stratified in vitro models whose absence was identified above, rather than as a therapy.

### 7.4. Toward Venous-Specific Rational Design

We propose that the field design for this indication rather than adapt general-purpose chronic wound hydrogels to it. Table 4 sets out the criteria that a hydrogel intended for venous ulceration would ideally satisfy simultaneously. The criteria fall into three families: performance beneath compression; behavior at the biological interface; and product viability. The list is deliberately framed as a reporting standard rather than as a specification: a system that fails a criterion may still be valuable, but the failure should be visible. Two entries carry particular weight. Net fluid absorption under interface pressure is the criterion most often inverted in practice, since the classical hydrogel donates moisture to a wound that is characteristically hyperexudative. And intrinsic or conferred antioxidant capacity with local iron chelation is the only entry that follows from an etiological mechanism specific to this disease rather than from chronic wound biology in general.

We are not aware of a published system that has been evaluated against all these criteria, and reporting against such a list would do more for translation in this indication than the addition of further novel chemistries. Table 4 and Table 5, the latter presented in Section 7.5, are indexed by criterion and by property, respectively, which suits a reader asking what a material must achieve. A materials scientist arriving from the other direction, asking what a given instrument can tell them about this indication, needs the same information indexed by technique. Supplementary Table S2 provides that cross-index, mapping each analytical method to the property it measures, the feature of venous pathobiology that makes the measurement necessary, and the therapeutic claim it supports or refutes. Several techniques appear there that are standard in hydrogel characterization but are rarely run under conditions that make them informative here, and the difference is usually the condition rather than the instrument. Vibrational spectroscopy illustrates the point. Fourier transform infrared and Raman spectroscopy are routinely used to confirm that a synthesis worked, and are rarely used to ask whether the product survives the medium for which it is designed. Acquiring spectra before and after a full bandage interval in pooled venous exudate would show directly whether crosslink and functional group signatures are retained, which no synthetic characterization can establish. One control is essential and rarely applied: exudate protein adsorbs to the network and contributes bands readily mistaken for changes in the network itself, so a protein-free comparison at matched pH and ionic strength is required before any loss is attributed to degradation.

X-ray photoelectron spectroscopy raises a related question with a sharper answer. It is the appropriate technique for the hydrogel–wound interface, since it reports elemental composition and oxidation state within the outermost few nanometres, which is where an antimicrobial payload, a surface-grafted chelator or a metal-containing component either is or is not presented to the wound. Bulk loading and surface availability are different quantities, and a construct can carry a substantial payload that never reaches the interface. The oxidation state information is the more useful half in this indication: whether silver is present as metallic or ionic species, and whether iron recovered from a used dressing is bound in the ferric state, are questions that distinguish a working mechanism from a plausible one, and neither can be answered by elemental quantification alone. The constraint is that the measurement requires ultra-high vacuum and therefore a dehydrated sample, so what is characterized is not the surface the wound encountered. Freeze-drying protocols preserve surface chemistry better than air drying but do not eliminate the problem, and any interpretation should state which was used. The practical consequence is that XPS is most informative when applied comparatively rather than absolutely, comparing a construct before and after exposure to exudate under identical preparation, where the artifact affects both measurements equally and the difference remains interpretable.

Beyond porosity, three observations available from the same instrument are more directly informative for this indication and are rarely reported. The first is the interfacial surface, imaged after exposure to exudate rather than after synthesis, which shows whether protein and cellular debris have occluded the network and closed the transport path on which absorption capacity depends. The second is cross-sectional gradient structure, since a construct that is dense at the wound face and open above will handle fluid differently from a homogeneous one of identical mean pore size, and the two are indistinguishable in a surface micrograph. The third is failure morphology after cyclic loading, which distinguishes a construct that has densified under compression, and therefore lost absorptive volume, from one that has fractured, and therefore lost integrity. These are different failure modes with different clinical consequences, and the rheological data alone cannot separate them. Coupling energy-dispersive X-ray analysis to the same acquisition adds the spatial distribution of a metal-containing payload, which answers whether an agent is dispersed through the network or aggregated at the surface, a distinction that governs release behavior and that bulk loading figures conceal. Both limitations point toward a method that the field has largely overlooked. X-ray micro-computed tomography reconstructs the network in three dimensions, and pore interconnectivity rather than pore size determines whether exudate transits the construct or pools at the interface, a topological property that no section can measure. A metal-containing payload has sufficient attenuation contrast to be mapped through the volume without labeling. Two features matter here in particular: hydrated imaging avoids the drying artifact that compromises electron microscopy, and the technique is non-destructive, so densification, fracture and progressive occlusion can be followed in the same specimen before and after cyclic loading rather than across separately prepared ones. Acquisition time rather than resolution is the constraint, which favors thorough characterization of a few candidate constructs rather than screening many.

### 7.5. Rheological and Mechanical Characterization Under Clinically Relevant Loading

Table 4 states what should be reported. It does not state at what values, and this omission is not incidental: the wound hydrogel literature has no agreed loading condition against which to characterize a material intended for this indication. The loading itself is well described in the compression literature and can be transferred directly. A bandage system targeting 40 mmHg at rest applies approximately 5.3 kPa at the ankle, and during ambulation an inelastic system produces working peaks of 60 to 80 mmHg, or 8 to 10.7 kPa, with the difference between working and resting pressure being the static stiffness index [134]. At a cadence near 1 Hz and 5000 to 10,000 steps daily, a gel worn across a weekly bandage interval experiences approximately 5 × 10^4^ loading cycles. This is the number that separates the venous indication from every other chronic wound: the calf pump loads and unloads the dressing tens of thousands of times between changes, in the malleolar region where conformability is most needed and where the gel has least mechanical support.

Three rheological measurements follow from this loading condition, and each is standard except in the conditions under which it should be run. A frequency sweep establishes whether the construct behaves as a solid across the timescale that matters, which is the bandage interval rather than the seconds typical of a laboratory sweep, so the low-frequency end determines the answer and is the end most often omitted. An amplitude sweep gives the yield stress, and a material whose yield stress falls below the applied normal stress will flow out from beneath the bandage whatever its storage modulus suggests; for sprayable and in situ-forming systems, the same sweep gives thixotropic recovery, which decides whether a deposited layer re-sets before the bandage is applied over it. Cyclic compressive loading is the measurement without an equivalent elsewhere in wound care, and it is the one for which no convention exists, which is why Table 5 specifies a cycle count rather than leaving it to be chosen. All three should be performed hydrated, at 37 °C and in a medium that resembles exudate rather than in buffer, since a network whose crosslinks are ionic or dynamic covalent will not exhibit the same moduli under the two conditions.

Swelling behavior carries the same problem in a more consequential form, since two independent errors compound: the absent load, already discussed, and the medium. Venous exudate is protein-rich, so adsorbed protein occludes the transport path and uptake falls below the buffer value and keeps falling, rather than reaching a plateau [120]; it carries divalent cations that displace the counterions of ionically crosslinked networks; and it is alkaline in the non-healing wound, which shifts the ionization of carboxylate and amine-bearing networks [130]. Distilled water reproduces none of this and phosphate-buffered saline reproduces only the ionic strength. Three values should therefore be reported rather than one: equilibrium swelling in buffer, which retains its comparative value across the literature; uptake in pooled or simulated venous exudate; and uptake in exudate under 5.3 kPa of sustained load, with the composition of any simulated fluid stated in full.

**Table 5:** Proposed engineering benchmarks for hydrogels intended for use beneath compression.

Property	Proposed Benchmark	Characterization Method
Storage modulus, G′	1 to 10 kPa	Frequency sweep at 1 Hz, 1% strain; matched to granulation tissue
Loss factor, tan δ	Below 0.3	Same sweep; above this, the material behaves as a paste rather than as a gel
Yield stress	Above 500 Pa	Amplitude sweep, G′/G″ crossover
Creep resistance	Under 20% thickness loss and under 5 mm lateral migration after 7 days at 5.3 kPa	Sustained compression on a wound bed phantom
Fatigue resistance	Above 80% retention of G′ after 5 × 10^4^ cycles between 5.3 and 10.7 kPa at 1 Hz	Cyclic compressive loading
Hydrated thickness over bony prominences	Below 3 mm	Interface pressure mapping with the construct in situ
Net fluid absorption under load	Positive at 5.3 kPa	EN 13726-1 with sustained load applied; no standardized loaded method currently exists
Thixotropic recovery (sprayable and in situ-forming systems)	Above 80% of G′ recovered within 60 s	Step amplitude sweep
High shear viscosity (sprayable systems)	Below 1 Pa·s at 100 s^−1^	Flow curve
Interfacial toughness on wet periwound skin	20 to 100 J/m^2^	Peel testing on a compromised skin model

Values are proposed as starting points for a reporting standard and require empirical refinement. Interface pressures and the static stiffness index derive from the compression literature [134]; EN 13726-1 is an existing standard for unloaded fluid handling [135]. All other values are inferred from soft tissue mechanics and from the loading conditions described in the text. G′, storage modulus; G″, loss modulus.

Thermal analysis addresses a question that arrives late and disqualifies constructs that performed well until then, since a wound hydrogel is a product before it is a material and must survive terminal sterilization, ambient storage and application outside a laboratory. Thermogravimetric analysis quantifies bound versus free water and the onset of decomposition, which sets the ceiling for any thermal sterilization route; differential scanning calorimetry gives the glass transition and melting behavior that determine dimensional stability across a distribution range. Both are more informative run on sterilized and aged samples against unprocessed controls than on freshly synthesized material, and chelators and antioxidants are frequently the least thermally robust component of an otherwise stable construct.

Characterization under these conditions is not technically demanding, and the parameters in Table 5 are within reach of any rheometry laboratory. We offer them as proposed starting values for a reporting standard rather than as established thresholds, since, with two exceptions, no such thresholds exist. The exceptions are informative. Interface pressures and the static stiffness index are settled quantities in the compression literature [134]. Fluid-handling capacity has a standard, EN 13726-1, but that standard measures absorption and moisture vapor transmission at rest [135]. There is no loaded variant, which means that the single most important property of a hydrogel in this indication, whether it still absorbs when a bandage is pressing on it, currently has no accepted method of measurement. Establishing one would be a more useful contribution to this field than a further novel chemistry.

The same reasoning transfers to superficial venous thrombosis, where the mechanical environment is gentler and the geometric constraints tighter. A dissolving microneedle array intended to deliver heparin across the stratum corneum over a phlebitic segment must cross an epidermis of roughly 100 μm without reaching the vein itself, which lies millimeters deeper, so the design target is dermal deposition with local diffusion rather than direct intravascular delivery. Needle lengths of 600 to 1000 μm, an array fracture force at least threefold above the per-needle insertion force, and a dissolution profile matched to a once-daily application interval are the parameters that would make such a patch specifiable. The reference dose is available: topical heparin gels in clinical use deliver 1000 IU/g [71,111,112], and a patch that cannot approach that local availability has no argument against the incumbent.

### 7.6. Methodological Priorities

Three methodological changes would alter the trajectory of this field more reliably than any material advance, and none of them requires new chemistry. The first is already available and largely unused: a core outcome set for venous leg ulcer research was agreed by international eDelphi consensus and published in 2024, covering healing, pain, quality of life, resource use and adverse events, with the patient rather than the limb or the ulcer as the preferred unit of analysis [132]. Every one of the four outcomes that the Cochrane hydrogel review found unreported falls within it [14], so adoption requires a decision rather than a method. The second is a minimum characterization and reporting standard for wound hydrogels, along the lines of Table 4 and Section 5.5, so that a trial result can be attached to a material rather than to a trade name. The third is a definitive trial, and since the network meta-analysis has already declared itself unable to say which comparison to prioritize [15], the selection must be driven by mechanistic rationale; on that basis, an antioxidant and iron-directed hydrogel is the defensible choice. Calling for such a trial in general terms has not moved this field, so Section 7.7 sets one out in enough detail to be costed and criticized.

### 7.7. A Trial the Field Could Design Today

We propose a two-stage program. The first stage establishes that the intervention engages its target; the second tests whether that engagement changes outcomes. Supplementary Table S3 gives the full specification, element by element, with the basis for each, and Supplementary Table S4 presents the sensitivity of the sample size to its assumptions.

The first stage is represented by a randomized mechanism engagement study in approximately 80 participants, allocated to the iron-directed hydrogel or to a vehicle hydrogel matched for fluid handling under load, under identical compression, for 12 weeks. The primary endpoint is biochemical rather than clinical: change from baseline at week 4 in wound-fluid labile iron and the MMP-9 to TIMP-1 ratio. Forty participants per arm detect a standardized difference of 0.7 with 80% power at a two-sided alpha of 0.05, allowing 25% for unusable exudate samples. Percentage area reduction at four weeks, which predicts healing at 24 weeks [133], is the clinical secondary endpoint. This stage is small and inexpensive; it answers the question this review identifies as unanswered, namely, whether venous specificity is a property of the material or an extrapolation; and it defines the chelator dose. The latter point is not administrative: collagen prolyl hydroxylase is iron-dependent (Section 3.3), so the therapeutic window must be established before a definitive trial is funded.

Enrichment starts with who is included in the trial. We would require an active ulcer in the gaiter region, an ankle-brachial pressure index of at least 0.80, and a Margolis index score of 1 or 2, meaning an ulcer area above 5 cm^2^, a duration beyond six months, or both [7]. Restriction to the slow-to-heal strata is the single most efficient design decision available here. In a secondary analysis of 802 participants across four trials, median time to healing rose from 40 days at a score of 0 to 57 days at a score of 1 and 86.5 days at a score of 2 [136]. Participants scoring 0 heal under compression alone; they contribute variance without contributing information about the dressing, which is one concrete mechanism by which compression dominance has masked dressing effects. A two-week run-in of protocolized compression before randomization, excluding ulcers whose area falls by more than 30%, removes the remainder of that group and identifies compression intolerance before rather than after allocation.

The choice of comparators follows from the same logic. Three arms run under identical compression: a simple non-adherent knitted viscose pad, a vehicle hydrogel matched for fluid handling under load, and the iron-directed hydrogel carried forward from stage one. The pad earns its place as the reference on two counts. It is the comparator against which the network meta-analysis produced its one moderate certainty signal [15], so it connects this trial to the only interpretable result the existing literature contains. It is also the cheapest defensible standard of care, which means that whatever increment is measured against it is the increment a payer will eventually be asked to fund. The vehicle arm is the element most often omitted and the one that makes the trial interpretable. Without it, a positive result cannot separate the chelating chemistry from a hydrogel matrix that simply handles exudate better than a pad, and that is the exact ambiguity which has made the existing literature uninformative. The primary comparison is the iron-directed hydrogel against the pad; the vehicle comparison is secondary and powered only for larger differences.

Setting the target effect means accepting that the smallest difference worth detecting is not the smallest difference that might exist. A functionalized hydrogel carries a unit cost premium over a knitted viscose pad, and an increment that does not at least offset the nursing time it displaces will not be adopted, whatever its *p* value. We therefore set the target at approximately ten additional percentage points of healing at 24 weeks. Against an assumed 45% in the enriched control population, this corresponds to a hazard ratio of 1.35 for time to healing, and detecting it with 90% power at a two-sided alpha of 0.05 requires 467 adjudicated healing events across the two arms of the primary comparison. VenUS 6 observed blind-assessed healing in 65% of randomized participants over up to 12 months [137]; we assume 60% here given the prognostic enrichment, which gives 390 participants per arm and 1170 in total. We would inflate this to 1260 and add a blinded, event-driven sample size re-estimation at the internal pilot, because the largest and most recent trial in this field accrued fewer healing events than its calculation assumed and lost power accordingly [137]. The corollary should be stated rather than buried: a trial of this size will not reliably detect a true difference of five percentage points, and we do not propose that it should, since a difference of that magnitude would not justify the cost of the intervention.

Controlling bias and cointervention is where this design departs most from its predecessors. Participants and treating nurses cannot be blinded, but outcome assessors can be, and VenUS 6 showed that blinded central photographic adjudication of healing works across 33 sites [137,138]. Compression is standardized to a single system in all arms, with a target of at least 40 mmHg at the ankle; interface pressure is verified with a subminiature transducer in a random tenth of applications; and receipt is logged at every visit. This matters more than any other single design feature. In VenUS 6, 71% of participants received a compression treatment other than the one allocated at some point during follow-up [137] and an uncontrolled cointervention sitting on the causal path will defeat a well-powered trial as readily as a small sample will. Departures from allocated compression should be handled through pre-specified estimands under both a treatment policy and a hypothetical strategy [139].

What gets measured, and what a result would mean, follow from the critique in Section 5.5. The primary outcome is time to blind-assessed complete healing of the reference ulcer over 12 months, analyzed by Cox regression with a shared frailty for center and with death treated as a competing risk. Secondary outcomes follow the core outcome set in full [132], including recurrence for 12 months after healing, which no trial in the Cochrane hydrogel review reported [14]. Wound fluid sampling continues from stage one in a nested subsample, and this is the component that allows a null result to be read: a material that failed to engage its target is a different finding from a target that does not matter, and no existing trial in this literature can distinguish the two.

Feasibility can be estimated rather than asserted, because a comparable trial has just finished. VenUS 6 randomized 637 participants across 33 United Kingdom sites over 38 months of recruitment, a rate close to 0.5 participants per site per month [137]. At that rate, 1260 participants require roughly 60 sites over 42 months. This is larger than any dressing trial yet conducted in venous ulceration and it is not deliverable by a single manufacturer or a single national program, but it is well within the reach of a European multicentre consortium and modest compared with cardiovascular trials addressing populations of comparable size. The obstacle is the funding structure described in Section 5.5 rather than the methodology. An internal pilot with pre-specified progression criteria on recruitment and on documented compression receipt would allow the design to be revised or abandoned before most of the cost is committed.

## 8. Conclusions

Hydrogels occupy an unusual position in venous disease. The mechanistic case for them is stronger here than in most chronic wound indications, because venous wound pathobiology presents a defined and chemically tractable set of targets, from catalytic iron and reactive oxygen species through protease excess to a senescent and dysregulated cell population. The clinical evidence, by contrast, is thinner than in almost any comparable field, and the gap between the two is the subject of this review.

Three conclusions follow from reading the material science against the clinical record. The first is that a mismatch of design intent sits at the center of the disappointing clinical results: the classical hydrogel donates moisture, the typical venous ulcer is hyperexudative, and the class of material for which clinical evidence has been gathered is not the class that the mechanistic argument actually recommends. The second is that the preclinical evidence base for this indication is, in a strict sense, absent, since no validated animal model of venous ulceration is in routine use and no in vitro system reproduces ambulatory venous hypertension, so that confident claims of venous specificity rest on extrapolation rather than on testing. The third is that the most tractable near-term development target may not be the chronic ulcer at all, but superficial venous thrombosis, where a topical gel route already has decades of clinical acceptance, the endpoints are symptomatic and measurable within weeks, and an accepted comparator exists.

The trial that would settle the question is specifiable today, in the sense that its population, comparators, endpoint, sample size and delivery model can all be written down using instruments and infrastructure that already exist (Section 7.7); what is missing is a funder whose remit covers a device question of this size.

None of this argues against hydrogels in venous disease. It argues for designing them against this disease specifically, characterizing them under the conditions they will actually encounter, and reporting them in terms that allow a clinical trial to be linked to a material rather than to a trade name. The chemistry needed to build such systems largely exists. What is missing is the discipline of aiming it at the right target and measuring whether it arrived.

## Figures and Tables

**Table 1:** Hydrogel polymer classes assessed against the venous wound, with the performance parameters not reported by the published literature.

Polymer Class	Typical Crosslinking	Documented Behavior Relevant to the Venous Wound	Key Limitation in the Venous Setting	Clinical Translation	Parameters Not Reported for This Class
Alginate [16]	Ionic coordination of divalent cations (egg-box)	Rapid gelation under mild conditions; high exudate uptake; progressive destabilization by Ca^2+^/Na^+^ exchange with exudate	Insufficient mechanical strength, uncontrolled degradation and susceptibility to chelating agents, the latter being incompatible with iron-chelating strategies	Marketed	Fluid handling under interface pressure; fatigue behavior; removal force on a dried wound bed
Chitosan [30,31]	Ionic and covalent; quaternized derivatives	Intrinsic antibacterial and hemostatic activity; cationic charge promotes tissue adhesion; quaternization broadens solubility	Requires acid processing or derivatization; non-selective antimicrobial action; mechanical instability under shear	Marketed and in trials	Fluid handling under interface pressure; wear time under compression
Hyaluronic acid [32,33]	Covalent (methacrylate, hydrazone)	Direct participation in repair signaling, with chains above ~1000 kDa being anti-inflammatory and degradation fragments being pro-inflammatory and pro-angiogenic	Depolymerized by hyaluronidase and reactive oxygen species, so the chronic wound shifts the population toward pro-inflammatory fragments	Marketed and in trials	Fluid handling under interface pressure; mechanical persistence under cyclic load
Collagen and gelatin [28]	Enzymatic and physical	Adhesion motifs supporting cellular attachment; degradable by intrinsic wound proteases	Batch variability and sourcing, viral and prion safety documentation; rapid proteolytic degradation in a catabolic wound	Marketed	Fluid handling under interface pressure; wear time; removal characteristics
Cellulose derivatives [37]	Physical and covalent	High absorbency; oxidized regenerated forms bind and sequester excess proteases	Bioactivity is derivative-dependent; unmodified forms contribute structure only	Marketed	Fluid handling under interface pressure; fatigue resistance across a bandage interval
Poly(vinyl alcohol), freeze–thaw [34,35]	physical (crystallite formation)	Crosslinker-free gelation simplifies toxicological assessment; cryogel porosity supports exudate uptake; performance depends on hydrolysis degree, molecular weight and freezing regime	Amorphous formulations donate moisture, which is mismatched to a hyperexudative wound; irradiation both crosslinks and sterilizes, requiring dose optimization per formulation	Marketed	Fluid handling under interface pressure; creep and migration under sustained load
Poly(ethylene glycol) based [16]	Covalent (radical, click)	Reproducible, mechanically tunable, free of batch-to-batch biological variation	Bioinert; contributes structure without signal, so functionalization is required for cell engagement	Preclinical and early trials	Fluid handling under interface pressure; wear time; removal from compromised periwound skin
GelMA and HAMA [16,38]	Photo-crosslinked	Retains biological recognition while gaining mechanical and processing control; degradation tunable across days to weeks by degree of substitution	Photoinitiator residues and polymerization radicals raise biocompatibility questions; no clinical data on venous ulceration	Preclinical	All four venous performance parameters; no venous-specific evaluation exists
Nanocomposite systems [39]	Matrix dependent	Metal and metal oxide fillers add antibacterial, catalytic and antioxidant function to a soft matrix	Each added component enlarges the characterization burden and the regulatory dossier; the silver precedent shows a strong antimicrobial rationale failing to translate in this indication	Preclinical	All four venous performance parameters; retention and long-term toxicology of particulate fillers

GelMA, methacrylated gelatin; HAMA, methacrylated hyaluronic acid. The final column is not a statement that these classes perform poorly against the listed parameters, but that they have not been evaluated against them. Fluid handling capacity under interface pressure, fatigue behavior under cyclic loading, wear time matched to a compression bandage interval and removal force from compromised periwound skin are the four parameters that determine performance beneath compression, and none is routinely reported in the wound hydrogel literature.

**Table 2:** Hydrogel payload classes, their mechanistic targets and the level of evidence reached in venous leg ulceration.

Payload Class	Mechanistic Target Addressed	Highest Level of Evidence in Venous Ulceration	Direction of That Evidence	Principal Formulation Obstacle
Silver (ionic, nanoparticulate)	Microbial burden, biofilm	Systematic review and network meta-analysis	No difference for healing; low-certainty signal over non-adherent dressings	Cytotoxicity at active concentrations
Iodine and honey preparations	Microbial burden, biofilm	Systematic review	No difference for healing	Exudate dilution shortens active period
Antimicrobial peptides, bacteriophage	Microbial burden, biofilm	Preclinical only	Not yet assessed	Proteolytic degradation in wound fluid
Photothermal and photodynamic systems	Microbial burden, biofilm	Preclinical only	Not yet assessed	Light delivery incompatible with compression
Growth factors	Arrested repair signaling	Randomized trials, other indications	Benefit in diabetic foot ulcer, not reproduced in venous ulcers	Short half-life under protease burden
Platelet-rich plasma	Arrested repair signaling	Systematic review	Uncertain, heterogeneous	Variable preparation, no standardization
Cells and skin equivalents	Cellular senescence, absent niche	Randomized trials	Benefit shown; adoption limited by cost	Viability, cold chain, manufacturing cost
Extracellular vesicles	Arrested repair signaling	Preclinical only	Not yet assessed	Isolation yield and potency assays
Antioxidants and nanozymes	ROS burden, lipid peroxidation	Preclinical only	Not yet assessed	Retention and toxicology of metal oxides
Iron chelators	Catalytic iron excess, ferroptosis	Absent in venous disease	Not yet assessed	Therapeutic window against iron requirement
Protease inhibitors, sacrificial substrates	Proteolytic imbalance	Randomized trials (non-hydrogel format)	Benefit suggested, evidence limited	Format is a matrix, not a hydrogel
Analgesic and anti-inflammatory agents	Pain, periwound inflammation	Randomized trials (foam format)	Symptomatic benefit	Format is a foam, not a hydrogel
Venoactive compounds	Capillary permeability, venous tone	Absent by topical route	Not yet assessed	Poor solubility and stratum corneum transport
Heparin and heparinoids	Local inflammation in SVT	Uncontrolled and dated clinical series	Symptomatic benefit reported	Transport of a large, charged macromolecule

**Table 3:** Evidence syntheses relevant to hydrogel dressings in venous leg ulceration.

Synthesis	Search Date and Scope	Studies and Participants	Comparisons Assessed	Principal Finding	Certainty	Outcomes Not Reported
Cochrane review of hydrogel dressings for VLU [13]	To May 2021; venous leg ulcers	Four RCTs in 10 articles; 272 participants (20 to 156 per trial)	Hydrogel versus gauze and saline, alginate, manuka honey, and hydrocolloid	Cannot be determined whether hydrogels are more effective for healing	Very low across all comparisons	Recurrence, quality of life, pain, cost
Cochrane review of antibiotics and antiseptics for VLU [98]	Venous leg ulcers	Multiple RCTs across agent classes	Honey, silver sulfadiazine, silver-impregnated dressings versus usual or standard care	No between-group difference in complete healing for any comparison	Low to very low	Recurrence, cost
Cochrane review of topical silver in infected chronic wounds [99]	Infected chronic wounds	Few short-duration trials	Topical silver versus alternatives	Trials too few and too short to support a recommendation	Insufficient	Healing beyond short follow-up, cost
Network meta-analysis of dressings and topical agents [14]	1985 to 2016; venous leg ulcers	78 studies, 7014 participants; 59 studies and 5156 participants in network	25 treatments compared	Signal favoring silver over non-adherent dressings; network uninformative overall, including for prioritizing a future trial	Moderate for that comparison; low for the network	Quality of life, pain, cost
Systematic review across DFU and VLU [95]	Diabetic foot and venous leg ulcers	Multiple RCTs across five dressing classes	Alginate, foam, hydrocolloid, hydrofiber and hydrogel versus basic contact dressings	Hydrogels more effective than basic contact dressings in DFU (RR 1.80, 95% CI 1.27 to 2.56); no difference for other comparisons	Moderate for the DFU finding	Recurrence, quality of life, cost

CI, confidence interval; DFU, diabetic foot ulcer; RCT, randomized controlled trial; RR, relative risk; VLU, venous leg ulcer. The final column lists outcomes that the synthesis reported as unavailable across its included trials.

**Table 4:** Proposed reporting criteria for hydrogels designed specifically for venous leg ulceration.

Domain	Criterion	Recommended Characterization
Fluid handling	Net absorption under 30 to 40 mmHg interface pressure	Fluid-handling capacity measured under load, not at rest
Mechanical persistence	Integrity and position retained over the bandage change interval	Fatigue testing under cyclic loading
Interface behavior	Conformal adhesion with atraumatic, ideally on-demand removal	Peel testing on compromised periwound skin models
Bulk and geometry	Minimal added bulk over the malleoli and the tibial crest	Interface pressure mapping with the construct in situ
Skin tolerance	Avoidance of sensitizers prevalent in this population	Formulation audit against contact allergen series
Etiological targeting	Antioxidant capacity with local iron chelation	ROS scavenging and iron binding assessed in venous wound fluid
Proteolytic control	Modulation of excess matrix metalloproteinase activity	Sacrificial substrate or specific inhibition, assayed in wound fluid
Manufacturability	Payload compatible with terminal sterilization and ambient storage	Stability data after sterilization and throughout the shelf life
Economic viability	Unit cost defensible against the nursing time displaced	Cost modeling against expected wear time

## Data Availability

No new data were created or analyzed in this study. Data sharing is not applicable to this article.

## Supplementary Materials

The following supporting information can be downloaded at: https://www.mdpi.com/article/10.3390/gels12090852/s1, Table S1: Proposed minimal preclinical battery for hydrogels claiming specificity for venous leg ulceration; Table S2: Analytical characterization methods indexed by technique, linked to the pathophysiological feature of venous leg ulceration that makes each measurement necessary; Table S3: Specification for a two-stage program evaluating an etiologically targeted hydrogel in venous leg ulceration; Table S4: Sensitivity of the Stage 2 sample size to the assumed treatment effect and event fraction.

## Abbreviations

The following abbreviations are used in this manuscript:

CEAP	Clinical, Etiological, Anatomical and Pathophysiological (classification)
CI	Confidence interval
DFU	Diabetic foot ulcer
EV	Extracellular vesicle
GelMA	Methacrylated gelatin
GMP	Good manufacturing practice
GPX4	Glutathione peroxidase 4
HAMA	Methacrylated hyaluronic acid
MMP	Matrix metalloproteinase
RCT	Randomized controlled trial
ROS	Reactive oxygen species
RR	Relative risk
SANRA	Scale for the Assessment of Narrative Review Articles
SVT	Superficial venous thrombosis
TGF-β1	Transforming growth factor beta 1
TIMP	Tissue inhibitor of metalloproteinases
VLU	Venous leg ulcer
