# Supplementary material for: Hydrogels in the Management of Peripheral Venous Disease: Bridging Material Design and Clinical Evidence
Source: Gels. 2026 Sep 19;12(9):852. doi: 10.3390/gels12090852 (PMC13606429; doi:10.3390/gels12090852)
Supplement: Supplementary file 1 [file gels-12-00852-s001.zip › gels-4524838-supplementary.pdf]

Supplementary Materials

Hydrogels in the Management of Peripheral Venous Disease: Bridging Material Design and Clinical Evidence

Table S1. Proposed minimal preclinical battery for hydrogels claiming specificity for venous leg ulceration.

Assay and conditions	Acceptance criterion or reported output
Mechanical performance under compression	
Cyclic compression fatigue: hydrated, 37 °C, simulated wound fluid, 5×10^4 cycles between 5.3 and 10.7 kPa at 1 Hz	≥80% retention of G′; under 20% thickness loss; net fluid uptake still positive after cycling
Creep under sustained load: 5.3 kPa for 7 days on a wound bed phantom	Under 20% thickness loss; under 5 mm lateral migration
Fluid handling per EN 13726-1, repeated with 5.3 kPa sustained load applied	Net absorption positive under load; both loaded and unloaded values reported
Behavior in venous exudate	
Degradation and payload release in pooled human VLU exudate, ≥10 donors, 37 °C, sampled at 0, 6, 24, 48, 72 and 168 h, fluid-to-gel ratio matched to the exudate output of the target wound class	Mass loss, G′ retention and cumulative release at each timepoint; integrity retained across a full bandage interval
Parallel arm in exudate treated with a broad-spectrum metalloproteinase inhibitor	Proportion of degradation attributable to proteolysis rather than hydrolysis
Release measured across at least three levels of MMP-9 activity, obtained by dilution of the pool	Monotonic relationship between protease activity and release rate, for any responsive-release claim
Equilibrium and kinetic fluid uptake in buffer, in pooled or simulated exudate, and in exudate under 5.3 kPa sustained load, sampled to 168 h	All three values reported; composition of any simulated fluid stated in full
FTIR or Raman spectra acquired before and after 168 h exposure to pooled exudate, with a protein-free control at matched pH and ionic strength	Retention of crosslink and functional group signatures; any loss attributed to the network rather than to adsorbed protein
Characterization of every exudate pool used: MMP-9 by gelatin zymography and immunoassay, MMP-9 to TIMP-1 ratio, total protein, pH, labile iron	Reported in full alongside the results obtained with that pool
Microbial burden	
Dual-species Staphylococcus aureus and Pseudomonas aeruginosa biofilm, ≥48 h maturation before treatment, continuous low shear in a drip flow reactor per ASTM E2647, simulated wound fluid or pooled exudate as medium, single application held for the intended wear time	≥3 $\log_{10}$ CFU reduction versus untreated control; vehicle-only comparator mandatory; clinical isolates tested alongside reference strains
Live/dead confocal imaging and biomass quantification of the treated biofilm	Viability distribution through biofilm depth, not surface counts alone
Cellular response	
Cytocompatibility and migration on senescent or stress-induced human dermal fibroblasts with confirmed loss of TGF-β1 responsiveness	Reported relative to a young control line of the same origin
Metabolic assay (MTT, CCK-8) paired with an orthogonal non-redox readout wherever the material is redox-active	Concordance between the two readouts demonstrated, or the discrepancy explained
Etiological targeting	
Labile iron in the test medium by calcein quenching or equivalent, at the delivered chelator dose	Demonstrated reduction relative to vehicle in venous exudate rather than in buffer

Assay and conditions	Acceptance criterion or reported output
Collagen prolyl hydroxylase activity at the delivered chelator dose	No significant inhibition at any dose proposed for clinical use

ASTM E2647 is the standard method for biofilm growth under low shear and continuous flow; EN 13726-1 is the existing standard for fluid handling measured at rest, for which no loaded variant currently exists. CCK-8, cell counting kit 8; CFU, colony-forming units; FTIR, Fourier transform infrared spectroscopy; G′, storage modulus; MMP, matrix metalloproteinase; MTT, 3-(4,5-dimethylthiazol-2-yl)-2,5-diphenyltetrazolium bromide; TGF-β1, transforming growth factor beta 1; TIMP, tissue inhibitor of metalloproteinases; VLU, venous leg ulcer. Acceptance criteria are proposed as a minimum standard for venous-specific claims and require empirical refinement; the loading conditions in the first group are derived in Section 7.5 and Table 5 of the main text.

Table S2. Analytical characterization methods indexed by technique, linked to the pathophysiological feature of venous leg ulceration that makes each measurement necessary.

**Analytical method**	**Measurable material property**	**VLU-specific challenge addressed**	**Expected therapeutic relevance**
Oscillatory rheology, frequency sweep	Storage and loss moduli, tan δ	The construct must remain solid-like across a seven-day bandage interval while conforming to an irregular wound bed	Too stiff a material distorts the pressure profile over bony prominences; too compliant a material extrudes from beneath the bandage
Oscillatory rheology, amplitude sweep	Yield stress, linear viscoelastic range, thixotropic recovery	Sprayable and in situ forming systems are applied and then immediately loaded by the bandage	Determines whether a thin deposited layer re-sets before compression is applied over it
Cyclic compressive loading (dynamic mechanical analysis)	Modulus retention and energy dissipation after approximately 5×10^4 cycles	The calf muscle pump loads and unloads the dressing tens of thousands of times between changes	The only mechanical demand unique to this indication; a construct characterized at rest has not been characterized for it
Compressive creep testing	Time-dependent deformation under sustained 5.3 kPa	Static interface pressure is applied continuously for the whole wear time	Lateral migration off the wound bed is a silent failure mode, invisible until the dressing is removed
Gravimetric fluid uptake under applied load	Net absorption capacity and uptake kinetics	Venous exudate is protein-rich and alkaline, neither of which the buffer reproduces, and the dressing absorbs while under sustained load	Reports three values: in buffer, in pooled or simulated exudate, and in exudate under 5.3 kPa; a construct saturating within a day macerates periwound skin for the rest of the interval
Moisture vapor transmission rate	Vapor flux across the construct	The dressing sits beneath a multilayer and partly occlusive bandage system	Measured in isolation, MVTR overstates evaporative capacity since the bandage is the rate-limiting barrier
Peel and lap-shear testing on compromised skin models	Interfacial toughness, removal force	Periwound skin is lipodermatosclerotic, frequently eczematous and often sensitized	Predicts skin stripping at removal, a common cause of discontinuation unrelated to efficacy
Interface pressure mapping with the construct in situ	Pressure distribution beneath the bandage	Retromalleolar hollows and the tibial crest already distort the Laplace pressure profile	Quantifies whether added bulk degrades the compression therapy to which the dressing is adjunct
Gelatin zymography and MMP immunoassay	MMP-9 activity, MMP-9 to TIMP-1 ratio	Proteolytic excess is a defining feature of the venous wound and the trigger for responsive systems	Distinguishes a genuinely protease-responsive system from one releasing on a fixed schedule
Rheometry in pooled exudate with inhibitor control	Degradation kinetics, mass loss	Degradation in buffer does not predict degradation in an actively proteolytic medium	Separates proteolytic from hydrolytic degradation, which changes the predicted wear time

Analytical method	Measurable material property	VLU-specific challenge addressed	Expected therapeutic relevance
Radical scavenging assays (DPPH, ABTS, ORAC)	Antioxidant capacity	Hemosiderin-derived Fenton chemistry sustains the local oxidative environment	Must be run in exudate rather than buffer, since exudate carries its own antioxidant capacity that a buffer measurement ignores
Calcein fluorescence and ferrozine assays	Labile iron pool, iron binding capacity	Local catalytic iron excess is the etiological feature specific to venous disease	Distinguishes true chelation from generic antioxidant activity; the measurement on which the venous rationale rests
ICP-MS or ICP-OES	Total iron sequestered in the recovered construct	Chelation claims in this literature are almost always qualitative	Quantifies how much iron is physically removed with the dressing, converting the claim into a stoichiometric one
FTIR spectroscopy	Functional group integrity, crosslink chemistry, conjugation density	Crosslinks are exposed to the pH, ionic load and protein content of venous exudate for a full bandage interval	Tracks chemical stability after exposure rather than after synthesis; requires a protein-free control at matched pH and ionic strength, since adsorbed exudate protein contributes bands mistaken for network change
Raman spectroscopy	Backbone and crosslink vibrational modes	Hydrated constructs can be examined without the lyophilization that alters network architecture	Complements FTIR by assessing the construct in the state in which it functions
XPS	Surface elemental composition and oxidation state within the outermost nanometres	Bulk payload loading and surface availability are different quantities; oxidation state determines whether silver or iron species are active at the interface	Confirms that a payload is presented at the wound interface rather than merely present; requires dehydration, so it is interpretable comparatively rather than absolutely
NMR spectroscopy	Confirmation and density of chelator conjugation	Spatial confinement depends on the chelator being tethered rather than free	Supports the extracellular restriction argument in Section 3.3; a released chelator makes a different claim from an immobilized one
Release kinetics by HPLC or UV in exudate	Payload release profile across 168 h	The bandage change interval defines the delivery window that must be covered	Burst release exhausts the payload before the interval ends, which no 24-hour release curve reveals
SEM, cryo-SEM and coupled EDX	Interfacial occlusion, cross-sectional gradient structure, failure morphology, spatial distribution of a metal-containing payload	Conventional drying makes reported pore size largely a record of ice crystal growth; exudate occludes the transport path on which absorption capacity depends	Distinguishes densification from fracture after cyclic loading, and dispersed from surface-aggregated payload, neither of which bulk figures reveal

Analytical method	Measurable material property	VLU-specific challenge addressed	Expected therapeutic relevance
X-ray micro-computed tomography	Three-dimensional network architecture, pore interconnectivity, depth-resolved payload distribution	Interconnectivity rather than pore size governs whether exudate transits the construct or pools at the interface, and no section can measure a topological property	Non-destructive and achievable hydrated, so the same specimen is imaged before and after cyclic loading or exudate exposure, removing between-sample variance from the comparison
TGA and DSC, with accelerated aging	Bound versus free water, decomposition onset, glass transition and melting behavior	The construct must survive terminal sterilization, ambient storage and distribution before it reaches a patient home	Sets the ceiling for thermal sterilization and indicates dimensional stability across a distribution range; most informative on sterilized and aged samples against unprocessed controls

This table is cross-indexed to Table 4 (reporting criteria by domain) and Table 5 (engineering benchmarks by property) of the main text, and to Table S1 (preclinical battery by assay), not a further independent standard. The techniques listed are standard in hydrogel characterization; what is specific to venous ulceration is, in almost every case, the condition under which each is performed. ABTS, 2,2′-azino-bis(3-ethylbenzothiazoline-6-sulfonic acid); DPPH, 2,2-diphenyl-1-picrylhydrazyl; DSC, differential scanning calorimetry; EDX, energy-dispersive X-ray spectroscopy; FTIR, Fourier transform infrared spectroscopy; HPLC, high performance liquid chromatography; ICP-MS, inductively coupled plasma mass spectrometry; ICP-OES, inductively coupled plasma optical emission spectrometry; MMP, matrix metalloproteinase; MVTR, moisture vapor transmission rate; NMR, nuclear magnetic resonance; ORAC, oxygen radical absorbance capacity; SEM, scanning electron microscopy; TGA, thermogravimetric analysis; TIMP, tissue inhibitor of metalloproteinases; VLU, venous leg ulcer; XPS, X-ray photoelectron spectroscopy.

Table S3. Specification for a two-stage program evaluating an etiologically targeted hydrogel in venous leg ulceration.

**Element**	**Specification**	**Basis**
STAGE 1 — MECHANISM ENGAGEMENT		
Design	Randomized, two-arm, parallel group, 12 weeks, single or a few centers	Establishes target engagement before committing to a definitive trial
Population	Adults with an active venous leg ulcer, ABPI ≥ 0.80, ulcer present ≥ 6 weeks	Standard VLU trial eligibility
Arms	Iron-directed hydrogel versus vehicle hydrogel matched for fluid handling under load; identical compression in both	Isolates the payload from the matrix
Primary endpoint	Change from baseline at week 4 in wound fluid labile iron and in the MMP-9 to TIMP-1 ratio	Tests the etiological mechanism directly rather than by extrapolation (Sections 3.1, 3.3)
Sample size	40 per arm, 80 in total: 32 per arm detect a standardized difference of 0.7 SD at 80% power, two-sided α 0.05, inflated 25% for unusable exudate samples	Conventional two-sample comparison of means
Secondary	Percentage area reduction at week 4; chelator dose tolerability; periwound skin integrity	PAR at 4 weeks predicts healing at 24 weeks; iron dependence of collagen prolyl hydroxylase makes dose finding necessary (Section 3.3)
STAGE 2 — DEFINITIVE EFFECTIVENESS TRIAL		
Design	Pragmatic, multicentre, three-arm, parallel group, 1:1:1, with internal pilot	Mirrors the delivered design of VenUS 6
Population	Adults with an active gaiter region ulcer, ABPI ≥ 0.80, and a Margolis index score of 1 or 2 (ulcer area > 5 cm^2, duration > 6 months, or both)	Prognostic enrichment removes the fast-healing stratum that contributes variance but no information on the dressing
Run in	Two weeks of protocolized compression before randomization; exclude ulcers with > 30% area reduction	Removes residual fast healers and identifies compression intolerance before allocation
Randomization	Central, computer generated, blocked, stratified by center, Margolis score (1 vs 2) and recurrent versus incident ulcer	VenUS 6 stratified on the same two ulcer-level predictors
Arms	A: simple non-adherent knitted viscose pad<br>B: vehicle hydrogel matched for fluid handling under load<br>C: iron-directed hydrogel carried forward from Stage 1	A is the cheapest defensible standard and the comparator behind the network meta-analysis signal; B is what makes a positive result interpretable
Compression	One system in all arms, target ≥ 40 mmHg at the ankle; interface pressure verified by subminiature transducer in a random 10% of applications; receipt logged at every visit	Converts the dominant cointervention from an uncontrolled confounder into a measured one; 71% of VenUS 6 participants received a compression treatment other than that allocated
Primary outcome	Time to complete healing of the reference ulcer, followed to 12 months	Avoids the information loss of a binary endpoint at a fixed time (Section 5.5)
Ascertainment	Blinded central photographic adjudication; date of healing is the date of the earliest photograph showing complete epithelial cover	Treatment blinding is impossible, outcome blinding is not

Element	Specification	Basis
Target effect	HR 1.35, equivalent to approximately 10 additional percentage points healed at 24 weeks against an assumed 45% in arm A	Set at the smallest increment that plausibly offsets the unit cost premium and the nursing time displaced, not at the smallest increment that might exist
Sample size	467 healing events in arms A and C; assuming 60% of randomized participants contribute an adjudicated event, 390 per arm and 1,170 in total, inflated to 1,260 (420 per arm)	Event-driven log-rank calculation; see Table S1 for sensitivity
Adaptation	Internal pilot with progression criteria on recruitment rate and documented compression receipt; blinded event-driven sample size re-estimation	VenUS 6 accrued fewer healing events than assumed and lost power as a result
Secondary outcomes	All five core outcome domains: healing, pain, quality of life, resource use, adverse events; plus recurrence to 12 months after healing; patient as unit of analysis	CoreVen international eDelphi consensus; all four outcomes missing from the Cochrane hydrogel review lie within it
Nested substudy	Wound fluid iron, ferritin, lipid peroxidation marker and MMP-9 / TIMP-1 at baseline, week 4 and week 12 in a subsample	Allows a null result to distinguish a material that failed to engage its target from a target that does not matter
Analysis	Cox regression with shared frailty for center, adjusted for stratification factors, age and mobility; death as competing risk; estimands for departures from allocated compression pre-specified under both treatment policy and hypothetical strategies	Follows current estimand guidance and the VenUS 6 analytic template
Multiplicity	Hierarchical testing: C versus A first, then C versus B; the vehicle comparison powered only for larger differences and reported as such	Preserves family wise error while retaining the mechanistic comparison
Feasibility	Approximately 60 sites over 42 months at the VenUS 6 recruitment rate of about 0.5 participants per site per month	Beyond a single manufacturer or single national program; within reach of a European consortium

ABPI, ankle brachial pressure index; HR, hazard ratio; MMP, matrix metalloproteinase; PAR, percentage area reduction; SD, standard deviation; TIMP, tissue inhibitor of metalloproteinases; VLU, venous leg ulcer. Sensitivity of the Stage 2 sample size to its assumptions is given in Table S4. Supporting references for the elements listed under Basis are cited in Section 7.7 of the main text.

Table S4. Sensitivity of the Stage 2 sample size to the assumed treatment effect and event fraction.

Panel A. 90% power, two-sided alpha 0.05.

Hazard ratio	Healing at 24 weeks, arm C (arm A = 45%)	Absolute difference	Events required (90% power)	Total N, three arms (f = 0.60)	Total N range (f = 0.55 to 0.65)
1.25	52.6%	+7.6 pp	845	2,112	1,950 to 2,304
1.30	54.0%	+9.0 pp	611	1,527	1,410 to 1,668
**1.35**	**55.4%**	**+10.4 pp**	**467**	**1,167**	**1,077 to 1,275**
1.40	56.7%	+11.7 pp	372	930	858 to 1,014
1.50	59.2%	+14.2 pp	256	642	591 to 699

Panel B. 80% power, two-sided alpha 0.05.

Hazard ratio	Events required (80% power)	Total N, three arms (f = 0.60)
1.25	631	1,578
1.30	457	1,143
**1.35**	**349**	**873**
1.40	278	696
1.50	191	480

Events are those required in the two arms of the primary comparison (arm A versus arm C) under a log-rank test. Total N assumes 1:1:1 allocation across three arms and an event fraction *f*, the proportion of randomized participants contributing an adjudicated healing event within 12 months. The proposed design is shown in bold: HR 1.35, 90% power, *f* = 0.60. Absolute differences are derived under an exponential survival assumption with 45% healing at 24 weeks in arm A. Figures are unrounded outputs of the calculation and would be inflated in a protocol as described in Section 7.7 of the main text. The full trial specification is given in Table S3.
